# Evaluating elexacaftor/tezacaftor/ivacaftor (ETI; Trikafta™) for treatment of patients with non-cystic fibrosis bronchiectasis (NCFBE): A clinical study protocol

**DOI:** 10.1371/journal.pone.0316721

**Published:** 2025-02-14

**Authors:** Colin E. Swenson, William R. Hunt, Candela Manfredi, Diana J. Beltran, Jeong S. Hong, Brian R. Davis, Shingo Suzuki, Cristina Barillá, Andras Rab, Cynthia Chico, Joy Dangerfield, Ashleigh Streby, Erin Barton, Elizabeth M. Cox, Arlene A. Stecenko, Adrianna Westbrook, Rebecca Kapolka, Eric J. Sorscher

**Affiliations:** 1 Division of Pulmonology, Allergy, Critical Care and Sleep Medicine, Department of Medicine, Emory University School of Medicine, Atlanta, GA, United States of America; 2 Division of Pulmonary, Asthma, Cystic Fibrosis and Sleep, Department of Pediatrics, Emory University School of Medicine and Children’s Healthcare of Atlanta, Atlanta, GA, United States of America; 3 Georgia Clinical & Translational Science Alliance, Emory University, Atlanta, GA, United States of America; 4 Division of Pulmonary, Allergy & Critical Care Medicine, University of Alabama at Birmingham Heersink School of Medicine, Birmingham, AL, United States of America; PLOS: Public Library of Science, UNITED KINGDOM OF GREAT BRITAIN AND NORTHERN IRELAND

## Abstract

**Background:**

Non-cystic fibrosis bronchiectasis (NCFBE) is a disease that exhibits dilatation of airways, airflow obstruction, persistent cough, excessive sputum production, and refractory respiratory infections. NCFBE exhibits clinical and pathological manifestations similar to key features of cystic fibrosis (CF) lung disease. In CF, pathogenesis results from dysfunction of the cystic fibrosis transmembrane conductance regulator (CFTR), and diagnosis is made by demonstrating elevated sweat chloride concentrations (typically ≥60 mEq/L), two CFTR mutations known to be causal, multi-organ tissue injury, or combination(s) of these findings.

**Objective:**

Based on a considerable body of evidence, we believe many patients with NCFBE have disease likely to benefit from drugs such as elexacaftor/tezacaftor/ivacaftor (ETI) that activate CFTR-dependent ion transport. ETI is currently prescribed solely for treatment of CF and has not been adequately tested or proposed for patients with NCFBE, many of whom exhibit decreased CFTR function. Accordingly, we are conducting a clinical trial of ETI in subjects carrying a diagnosis of NCFBE.

**Methods:**

Participants will exhibit one disease-causing CFTR mutation and/or sweat chloride measurements of 30–59 mEq/L. Cutaneous punch biopsy or blood samples will be obtained for iPS cell differentiation into airway epithelial monolayers–which will then be tested for response to ETI. Each patient will be given CFTR modulator treatment for approximately four weeks, with monitoring of clinical endpoints that include FEV_1_ (forced expiratory volume in one second), sweat chloride, quality of life questionnaire, and weight. The study will evaluate response of patients with NCFBE to ETI, and test usefulness of iPSC-derived airway epithelial monolayers as a novel in vitro technology for predicting clinical benefit.

**Trial registration:**

This trial is registered at clinicaltrials.gov (Identifier: NCT05743946. Date: 02/23/2023).

## Introduction

### Objectives

#### ‘Lead in’ study: Evidence for diminished CFTR activity among patients with NCFBE

In order to gain information regarding numbers of individuals with NCFBE who may be eligible for the ‘main’ study (see below), patients at Emory followed with bronchiectasis (who do not exhibit clinical manifestations adequate for a diagnosis of cystic fibrosis) will be asked to consider participating in a ‘lead in’ study that will determine CFTR genotype and sweat chloride level for each subject. A separate consent form will be utilized for the ’lead in’ study. Individuals who exhibit a single CF-causing mutation in CFTR and/or sweat chloride 30–59 mEq/L will be approached about their interest in reviewing the consent form for the ‘main’ study (“iPSC derivation and in vivo ETI treatment for 4 weeks”; see following section). Up to 200 subjects will be included in the ‘lead-in’ study.

### ‘Main’ study: iPSC derivation and in vivo ETI treatment for 4 weeks

We propose a clinical trial of subjects with a diagnosis of NCFBE. Participants will exhibit one disease-causing CFTR mutation and/or sweat chloride measurements of 30–59 mEq/L. Each patient will be given ETI for approximately four weeks. We will monitor clinical endpoints that include FEV_1_, sweat chloride, quality of life questionnaire, and weight. We will also collect cutaneous punch biopsy material or blood samples from subjects so that iPS cells can be differentiated into airway epithelial monolayers and tested for response to ETI.

## Background

Non-cystic fibrosis bronchiectasis is a clinical syndrome characterized by abnormal dilatation of the airways, airflow obstruction, persistent cough, excessive sputum production and recurrent lung infections. In terms of pathophysiology, airway dilatation and other features are associated with impaired mucociliary transport and failure to adequately clear bacteria and mucinous secretions from the respiratory tract. These events contribute to persistent infection, inflammation, and further airway damage, leading to diminished lung function, with the possibility of pulmonary failure and death. The pathogenesis of non-CF bronchiectasis is complex, poorly understood, and is likely to vary depending on the underlying etiology and important modifying factors [1–3].

NCFBE is clinically and pathologically similar to certain features of cystic fibrosis lung disease. For example, like CF, NCFBE pulmonary injury is characterized by pronounced bronchiectasis, airway architectural damage, mucus accumulation, chronic lung infection and persistent neutrophilic infiltrates [2–6]. In CF, disease is caused by generalized dysfunction of the cystic fibrosis transmembrane conductance regulator. By convention, sweat chloride levels in NCFBE are <60 mEq/L. NCFBE is therefore often considered a diagnosis of exclusion–if criteria are inadequate to establish a diagnosis of “cystic fibrosis”, NCFBE may instead be entertained. Patients with NCFBE are not approved for ETI, and do not have access to the drug. Based on a considerable body of evidence, we believe a substantial number of patients with NCFBE have disease likely to exhibit clinical benefit from drugs such as ETI that activate CFTR-dependent ion transport. Such a notion has not been adequately tested or proposed previously.

In a subset of individuals with NCFBE, pathogenesis is likely due–at least in part–to CFTR deficiency, originating from inherited factors (i.e., asymptomatic CF carrier status, which occurs in approximately 1 of 30 US Caucasians and leads to ~50% decrease in CFTR mRNA and protein), acquired factors (e.g., due to chronic airway inflammation, infection, hypoxia, or toxin exposure), or a combination of these. For example, in patients with chronic airway infection and inflammation, or past toxin (e.g. nicotine) exposure, CFTR activity has been suggested to be negatively impacted–resulting in defects in anion secretion, airway surface liquid depletion and blunted mucociliary transport [7, 8]. Furthermore, CFTR mutation carriers, with an estimated half the level of functional CFTR compared to the general population, are at increased risk for a range of adverse conditions, including NCFBE [9–12]. Moreover, in a study of 122 individuals with NCFBE and normal sweat chloride concentrations, 22 (18%) were found to have one CFTR mutation and abnormal CFTR function in respiratory tract epithelium that was intermediate between healthy controls and those with classical CF [13].

### Considerations regarding NCFBE

The incidence of NCFBE is increasing worldwide. Previously classified as a rare or orphan disease, the diagnosis has increased by 40% in the past several years [1] and the number of adults with bronchiectasis in the United States is estimated at 350,000–500,000 [14]; i.e., much higher than the number of patients with CF (which amount to 30,000–40,000). Unfortunately, there are no curative therapies or medications specifically approved to address fundamental mechanisms that underlie NCFBE.

ETI is registered for patients with CF carrying at least one copy of the common F508del variant or a number of other CFTR abnormalities. The drug is a combination of three CF therapeutic compounds: elexacaftor, tezacaftor, and ivacaftor, that allow CFTR to function more effectively. Patients with common forms of CF typically exhibit robust pulmonary benefit from ETI within several days of initiating treatment [6, 15, 16].

ETI is not prescribed or approved for NCFBE and has not been considered of benefit for patients with NCFBE. We hypothesize that a drug combination such as ETI, which markedly activates both wild type and mutant CFTR, will enhance mucociliary clearance and should improve respiratory function (FEV_1_) in a subset of patients with NCFBE. Even if one in ten patients with NCFBE shows significant improvement in FEV_1_ following ETI, this would constitute a major discovery and provide new hope for tens of thousands of individuals in the US with an otherwise untreatable and lethal lung disease.

### Emerging approaches to identify patients with CF or other respiratory conditions most likely to exhibit clinical response to ETI

The nature and extent of in vitro model systems that predict pulmonary responsiveness to ETI are being evaluated by several laboratories, including our own. Reprogramming of adult somatic cells into iPSCs is a powerful approach that holds promise for regenerative and personalized medicine. This system presents potential advantages over classical in vitro cell-based and other models, and has been useful for generating efficient, high-performance tools for clinical/translational research and drug development [17]. From a translational perspective, the use of patient-derived specimens may allow a comparative test of drug efficacies in vitro and therapeutic responses at an individual level, as recently evaluated by a number of investigators, including those participating in the current project [18–25].

### Summary

**Fig 1:**
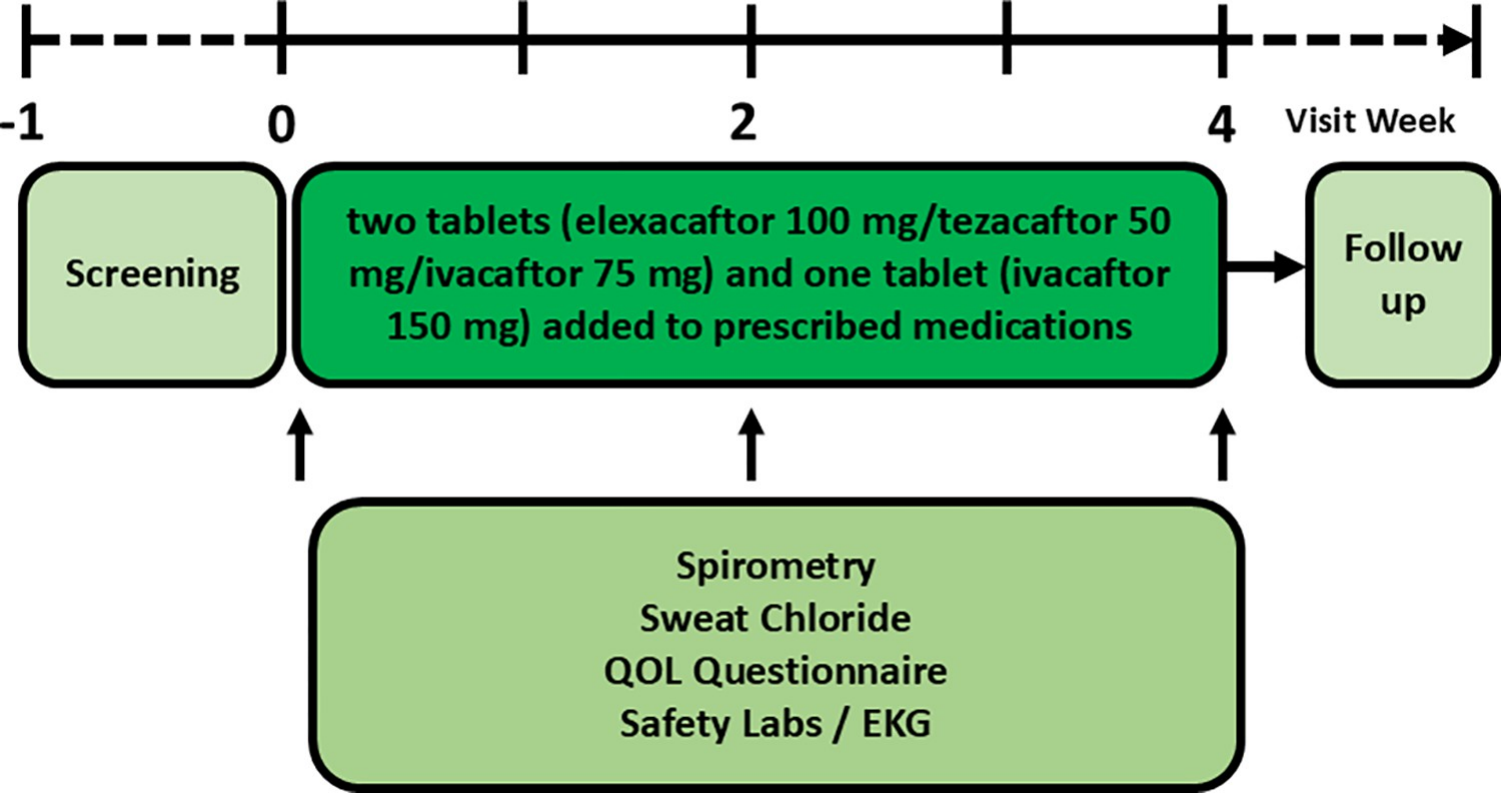
Summary of study design.

We believe many individuals with NCFBE will respond to stimulation of CFTR function in the airway, including patients with sweat chloride values that are normal or only mildly elevated (i.e., the latter indicating systemically diminished CFTR activity). The present study will comprise an open-label, single center trial of orally administered elexacaftor, tezacaftor and ivacaftor that will enroll 30 patients with NCFBE. Study subjects will have one known CFTR mutation and/or mildly elevated sweat chloride measurements (i.e., 30–59 mEq/L). **Fig 1** summarizes trial design, including conventional trial endpoints. Patients with a diagnosis of CF will be excluded (see below). The study will specifically and prospectively test iPS cells taken from patients with NCFBE to determine in vitro thresholds for predicting CFTR rescue in vivo. Using iPS cells differentiated to exhibit a respiratory epithelial phenotype, we will determine whether the emerging technology can be used to predict FEV_1_ response among individuals with NCFBE who receive ETI.

## Materials and methods

This protocol was approved by the Emory Institutional Review Board (IRB#00004736), with written consent obtained.

### “Main” study endpoints (Table 1)

- Primary Endpoints: iPSC-derived evidence of ETI response in vitro and pulmonary function testing of FEV_1_ (forced expiratory volume in one second) following ETI administration in vivo
- Secondary Endpoints: sweat chloride, NCFBE-directed quality of life tool, weight gain, safety laboratory evaluation/EKG

**Table 1:** Primary and secondary endpoint summary.

OBJECTIVES	ENDPOINTS	JUSTIFICATION FOR ENDPOINTS
Primary		
To determine whether iPS cells differentiated to airway epithelia—and treated with ivacaftor/tezacaftor/elexacaftor in vitro*—*can predict clinical responsiveness to ETI in vivo. To evaluate FEV_1_ clinical responsiveness to ETI in vivo.	• Functional and biological correction of CFTR expressed in iPS cells as judged by short circuit current measurements in monolayers, and western blot analysis. • Forced expiratory volume in one second (FEV_1_).	• These in vitro tests represent standard functional and biochemical analyses that address CFTR rescue, with direct relevance to the in vivo situation. • FEV_1_ is a conventional endpoint and provides a direct measurement of patient health benefit among individuals with NCFBE.
Secondary		
To verify safety and further establish efficacy of ETI in enrolled patients.	• Sweat chloride test • Quality of life survey • Standard laboratory tests and other measurements, as well as history and physical examination	• Provides supportive information regarding study intervention effect using in vivo measures known to indicate CFTR rescue• A well-established tool for monitoring patient benefit• Confirm safety of the FDA-approved drug

### Study intervention/investigational agent

Subjects will be given the Study Drug elexacaftor 100 mg/tezacaftor 50 mg/ivacaftor 75 mg (2 pills once daily in the morning) and ivacaftor (150 mg) once daily in the evening, as the FDA-registered agent, ETI. Dose and schedule for this open label, single arm, unblinded trial will be identical to what has already been FDA-approved for effective treatment of CF. The research pharmacy will be used since ETI is not on label for NCFBE.

### Procedures involved

#### Human subjects involvement

The study will examine iPSC-derived airway epithelial monolayers (an in vitro analytic tool) as a predictive means for identifying individuals with NCFBE most likely to show respiratory benefit from ETI. The airway cells will be generated using blood and/or skin samples taken from participants. Enrollment is planned at Emory University. Patients in the trial will have one disease-causing CFTR variant and/or modestly elevated sweat chloride values (30–59 mEq/L), but without clinical findings sufficient for a diagnosis of cystic fibrosis.

Human subject participation in the ‘main’ study will consist of a screening day (-28 to -1), day 1 (+/-2), day 7 (+/- 2) [phone call visit], day 14 (+/-2), day 28 (+/- 2) and day 56 (+/-2) follow up (wash-out). The visits will include patient history, physical exam, safety laboratory assessments (renal and liver panels, CBC/Diff, urinalysis), serum and urine pregnancy testing, CFTR genotype, spirometry, O_2_ saturation, sweat chloride test, blood sample collection and/or cutaneous punch biopsy for iPSC derivation, EKG, drug accountability, questionnaire for quality of life (QOL) assessment, concomitant medication review, and adverse event reporting.

All participants will provide written informed consent, with separate forms used for screening (‘lead-in’ study) and treatment (‘main’ study).

### “Main” study inclusion and exclusion criteria

We anticipate the screening and enrollment visit for the ‘main’ study will require 4–6 hours to complete the informed consent, medical history, O_2_ saturation, concomitant medication review, physical exam, safety laboratory assessments (CBC/Diff, BUN, creatinine/LFT chemistry panel, and urinalysis), EKG, CFTR genotyping, questionnaire, serum pregnancy test for females capable of pregnancy, sweat chloride test, and spirometry.

### Inclusion criteria

- Provision of signed and dated informed consent form
- Stated willingness to comply with all study procedures and availability for the duration of the study
- Male or Female age ≥18
- Radiologic and other clinical evidence leading to a diagnosis of NCFBE
- 1 CF-causing mutation and/or sweat chloride measurement ≥ 30 mEq/L and < 60 mEq/L
- Able to perform spirometry meeting ATS criteria for acceptability and repeatability, and FEV_1_ 40–90% predicted. Clinically stable in the past 4 weeks with no evidence of bronchiectasis exacerbation (prior to Screening AND Day 1).
- Willingness to use at least one form of acceptable birth control, including abstinence or condom with spermicide. This will include birth control for at least one month prior to screening and agreement to use such a method during study participation for an additional four weeks after the last administration of Study Drug. For postmenopausal or other women who are without the possibility of becoming pregnant, this requirement may be waived if a physician deems the study subject as “N/A”.
- Ability to take ETI
- Agreement to adhere to all current medical therapies as designated by the study physician

### Exclusion criteria

- Diagnosis of cystic fibrosis
- Documented history of drug or alcohol abuse within the last year
- Pulmonary exacerbation or changes in therapy for pulmonary disease in the 4 weeks prior to screening
- Listed for lung or liver transplant at the time of screening
- Cirrhosis or elevated liver transaminases > 3X upper limit of normal
- Pregnant or breastfeeding
- Inhibitors or inducers of CYP3A4, including certain herbal medications and grapefruit/grapefruit juice, or other medicines known to negatively influence ETI administration
- History of solid organ transplant
- Active therapy for non-tuberculosis mycobacterial infection or any plan to initiate non-tuberculosis mycobacterial therapies during the study period
- Known allergy to ETI
- Treatment in the last 6 months with an approved CFTR modulator
- Any other condition that, in the opinion of the lead investigators, might confound results of the study or pose an additional risk from administering Study Drug
- Treatment with another investigational drug or other intervention within one month prior to enrollment, throughout the duration of study participation, and for an additional four weeks following final drug administration.
- Evidence of cataract/lens opacity determined to be clinically significant by an ophthalmologist at or within 3 months prior to the Screening Visit

Study status and timeline. The study received Institutional Review Board (IRB) approval on December 19, 2022. Participant recruitment is ongoing at the time of this report. The lead-in study began enrolling on April 12, 2023, with participants joining the main study (or treatment component) on April 18, 2023. It is anticipated that final study participants will be enrolled by December 30, 2025, with treatment and data collection to be finalized in June 2026. Analysis and final results are expected by December 2026.

### Expected outcomes

Please note that based on past experience conducting clinical trials, we do not have concerns regarding our ability to perform the patient-oriented study described here, or to propagate and characterize iPS cells [19–21, 26]. The ‘main’ study protocol will evaluate clinical benefit of ETI in patients with NCFBE. In addition, the study will furnish one test of our hypothesis that thresholds of CFTR activity in differentiated iPS cells in vitro can predict benefit following ETI in vivo. If a strong correlation can be established between ETI response in vitro and in vivo (e.g., FEV_1_), the results will provide the first evidence regarding usefulness of in vitro models for predicting clinical efficacy in this disease setting.

### Statistical analysis plan

Goals of the “main” study are to determine the clinical response to ETI and whether in vitro measurements using iPS cell-derived airway monolayers predict ETI effectiveness. Study leadership, together with physician assistants and coordinators, will assess clinical findings. Primary outcomes comprise the ability of iPSC-derived monolayers to predict respiratory benefit from ETI after 4 weeks of in vivo treatment. Primary analysis will include an estimate of clinical response rate of ETI at week 4 of treatment and a 95% confidence interval using the exact method proposed by Blaker [27]. A responder is defined as any subject with an improvement, from baseline, in FEV_1_ > 5% predicted. FEV_1_ will also be considered continuously. A two-sided 0.05 alpha level paired t-test comparing the baseline and week 4 FEV_1_ measurements, along with the test and 95% associated confidence interval, will be evaluated.

In this study, if at least 15% of subjects meet the definition of responder, we will view this as initial evidence of a favorable result. Overall response rate will be accompanied by an exact 95% confidence interval. To determine the role of iPS in vitro response to ETI, we will compare the Δ lsc (change in short circuit current) among ETI responders and ETI non-responders using an ANOVA test. Additionally, we will correlate the Δ lsc obtained from in vitro experiments with the change in FEV_1_% predicted (post minus pre) using Spearman’s rank-order correlation with an associated 95% confidence interval. We will also test whether the iPS response reaches a CFTR functional threshold of 30% wild-type levels (a magnitude of activity in primary airway epithelial cells viewed as potentially relevant to clinical benefit). In a sensitivity analysis, we will replicate the FEV_1_ analysis with sweat chloride responses and determine the in vivo response rate.

Secondarily, we will serially evaluate clinical outcomes of interest prior to and after the start of therapy. Change in clinical measurements (in addition to spirometry–sweat chloride, weight/BMI, quality of life) will be monitored. A complementary analysis evaluating the percentage of participants with ≥15 mmol/L decline in sweat chloride will also be reported. With a previously estimated 9.7 mmol/L standard deviation of the change in sweat chloride for successful CF modulator trials [28, 29], an anticipated 6% of responders would be expected by chance alone. Therefore, the treatment response prevalence will be compared to a reference rate of 6%. For longitudinal FEV_1_ through day 28 and for secondary endpoints (absolute change in sweat chloride, QOL questionnaire, and weight/BMI from baseline through day 28), means at each time point and changes over time will be summarized along with corresponding confidence intervals estimated by mixed effects models to account for within-subject correlation.

Changes in FEV_1_ following treatment with ETI will be correlated with secondary clinical outcome measures. A non-linear Spearman’s correlation coefficient will be estimated for each measure paired with iPS cell responses in vitro, with corresponding fitted curves displayed graphically along with coordinates for participant’s paired in vivo and in vitro responses.

For other outcomes of interest including sweat chloride levels, quality of life, and weight/BMI, we will also apply a mixed effect model to examine the change in these measures over the study duration. Models will include a subject-specific intercept, to account for variability among subjects prior to the start of the treatment, and a categorical variable for time (baseline, day 14, day 28, day 56). A Dunnet’s post-hoc comparisons procedure will be used to determine which time points demonstrated a significant change from baseline. Using the same mixed effects models, we will include Δ Isc as predictors of in vivo improvement. We will also test the interaction between time and Δ lsc to determine when in vitro response is most associated with change in clinical response. Adverse events and other safety endpoints will be tabulated and reported using counts and percentages or means and standard deviations, as appropriate.

Because clinical parameters may vary among patients prior to the start of therapy, we will also approach the analysis using a modified N of 1 design configured for the purpose of comparing iPS studies in contrast to the aggregate analysis described above. Using the framework of single subject research, we will evaluate therapeutic response in individual subjects by an interrupted time series (ITS) method. Briefly, in the absence of an independent control group, ITS analysis is a quasi-experimental design in which each subject acts as their own control and is followed serially in time prior to and after an interruption (in this case, the start of treatment). If the treatment has causal impact, the post-intervention time series will have a different level or slope than the pre-intervention series. Time series models also have the power to test and correct for possible cyclical patterns and outliers. Using segmented regression analysis, we will conduct ITS models for each of the subjects enrolled in the trial. A significant change in slope from the pre-treatment period will indicate a significant treatment effect. ITS models will be constructed for each outcome of interest where historical and pre-treatment data are available. The change in slope will also be correlated with the in vitro response using Spearman’s rank-order correlation coefficient.

All analyses will be conducted using the intention to treat principal, where all subjects will be included in analysis regardless of how long they took the study medication. Statistical significance will be assessed at the 0.05 level and analyses conducted using SAS v. 9.4 (SAS Institute; Cary, NC).

## Supporting information

S1 Checklist SPIRIT checklist. (PDF)

S1 File Full protocol. (PDF)

S2 File Informed consents. (PDF)

S1 Fig SPIRIT schedule of assessments. (TIF)
