# Supplementary material for: Evaluating elexacaftor/tezacaftor/ivacaftor (ETI; Trikafta™) for treatment of patients with non-cystic fibrosis bronchiectasis (NCFBE): A clinical study protocol
Source: PLoS One. 2025 Feb 14;20(2):e0316721. doi: 10.1371/journal.pone.0316721 (PMC11828409; doi:10.1371/journal.pone.0316721)
Supplement: S1 File — (PDF) [file pone.0316721.s002.pdf]

PROTOCOL TITLE: Evaluating Trikafta for the treatment of patients with non-cystic fibrosis bronchiectasis (NCFBE)

EXTERNAL (NON-EMORY) COLLABORATORS

Brian R. Davis, PhD

Associate Professor of Molecular Medicine, Center for Stem Cell Research, Brown Foundation Institute of Molecular Medicine, University of Texas Health Science Center at Houston, Houston, TX

The University of Texas Health Sciences Center IRB will review Dr. Davis' human subjects research activities.

PRINCIPAL INVESTIGATORS:

Eric Sorscher, Professor, Department of Pediatrics

Randy Hunt, Associate Professor, Department of Medicine

Arlene Stecenko, Associate Professor, Department of Pediatrics

VERSION: 14Sept2022.v1

FUNDING SOURCE: Marcus Foundation, Inc

REVISION HISTORY

Revision #	Version Date	Summary of Changes

Table of Contents

1. Study Summary 4
2. Objectives 5
3. Background 5
4. Study Endpoints 8
5. Study Intervention/Investigational Agent 9
6. Procedures Involved 9
7. Statistical Analysis Plan 13
8. Data and/or Specimen Banking 17
9. Sharing of Results with Participants 18
10. Study Timelines 20
11. Inclusion and Exclusion Criteria 20
12. Population 21
13. Vulnerable Populations .... **Error! Bookmark not defined.**
14. Local Number of Participants 21
15. Recruitment Methods 22
16. Withdrawal of Participants 23
17. Risk to Participants 24
18. Potential Benefits to Participants 26
19. Compensation to Participants 26
20. Data Management and Confidentiality 27
21. Plans to Monitor the Data to Ensure Safety of Participants and Data Integrity 27
22. Provisions to Protect the Privacy Interest of Participants 33
23. Economic Burden to Participants 34
24. Informed Consent 34
25. Setting 35
26. Resources Available 35
27. Multi-Site Research When Emory is the Lead Site .... **Error! Bookmark not defined.**
28. References 36
29. Protocol Checklist 40

1. Study Summary

**Project Title**	Evaluating Trikafta for the treatment of patients with non-cystic fibrosis bronchiectasis (NCFBE)
**Project Design**	The study will examine iPSC-derived airway epithelial monolayers (an in vitro analytic tool) as a predictive means for identifying individuals with NCFBE most likely to show respiratory benefit from Trikafta™. As part of the protocol, we will perform an open-label clinical trial of orally-administered Trikafta™ in subjects with NCFBE.
**Primary Objective**	iPSC-derived evidence of a Trikafta™ response and pulmonary function testing of FEV_1 (forced expiratory volume in one second) following Trikafta™ administration in vivo
**Secondary Objective(s)**	Sweat chloride, NCFBE-directed quality of life tool, weight gain, safety laboratory evaluation/EKG
**Research Intervention(s)/Interactions**	Elexacaftor 100 mg/tezacaftor 50 mg/ivacaftor 75 mg (2 pills once daily in the morning) and ivacaftor (150 mg) once daily in the evening, as the FDA-registered agent, Trikafta™. Dose and schedule will be identical to what has already been FDA-approved for effective treatment of CF.
**Study Population**	Individuals with a diagnosis of NCFBE, who have a single cystic fibrosis transmembrane conductance regulator (CFTR) disease-causing mutation and/or modestly elevated sweat chloride values (30-60 mEq/L), but without clinical findings sufficient for a CF diagnosis
**Sample Size**	30
**Study Duration for individual participants**	4 months
**Study Specific Abbreviations/ Definitions**	CF - cystic fibrosis<br>CFTR - cystic fibrosis transmembrane conductance regulator

	FEV_1 -Forced expiratory volume in one second<br>iPS cells – induced pluripotent stem cells<br>NCFBE - non-cystic fibrosis bronchiectasis
**Funding Source (if any)**	The Marcus Foundation, Inc

2. Objectives

'Lead in' study: Incidence of diminished CFTR activity among patients with NCFBE

In order to gain information regarding numbers of individuals with NCFBE who may be eligible for our 'Main' study trial, patients at Emory followed with a diagnosis of bronchiectasis (who do not have clinical criteria sufficient for a diagnosis of cystic fibrosis) will be asked to consider participating in a 'lead in' study that will determine the subject's CFTR genotype and sweat chloride. A separate consent form will be utilized for the 'lead in' study. Subjects who exhibit a single CF-causing mutation in CFTR and/or sweat chloride 30-60 mEq/L will be approached about their interest in reviewing the consent form for the 'main' study ("iPSC derivation and in vivo Trikafta™ treatment for 4 weeks"; see following section). Up to 300 subjects may be included in the 'lead-in' study.

'Main' study: iPSC derivation and in vivo Trikafta™ treatment for 4 weeks

We propose a clinical study of subjects with a diagnosis of NCFBE. Subjects will exhibit one disease-causing CFTR mutation and/or sweat chloride measurements of 30-60 mEq/L. Each patient will be given Trikafta™ for approximately four weeks. We will monitor clinical endpoints that include FEV_1, sweat chloride, quality of life questionnaire, and weight. We will also collect cutaneous punch biopsy material from each subject so that iPS cells can be differentiated into airway epithelial monolayers and tested for response to Trikafta™.

3. Background

Non-cystic fibrosis bronchiectasis is a clinical syndrome characterized by abnormal dilatation of the airways, airflow obstruction, persistent cough, excessive sputum production and recurrent lung infections. In terms of pathophysiology, airway dilatation and other features are associated with impaired mucociliary clearance and failure to adequately clear bacteria and mucus secretions from the airways. These events contribute to persistent infection, inflammation, and further progressive airway damage, leading to diminished lung function and eventually respiratory failure and death. The pathogenesis of non-CF bronchiectasis is complex, poorly understood, and is likely to vary depending on the underlying etiology and important modifying factors (Maselli, Amalakuhan et al. 2017, Chalmers, Chang et al. 2018, Flume, Chalmers et al. 2018).

NCFBE is clinically and pathologically similar to certain features of cystic fibrosis lung disease. For example, like CF, NCFBE pulmonary injury is characterized by pronounced bronchiectasis, airway

architectural damage, mucus accumulation, chronic lung infection and persistent neutrophilic infiltrates (Maselli, Amalakuhan et al. 2017, Chalmers, Chang et al. 2018, Manfredi, Tindall et al. 2019, Cabrini, Rimessi et al. 2020). In CF, because of generalized dysfunction of the cystic fibrosis transmembrane conductance regulator, the diagnosis is made by demonstrating elevated sweat chloride concentrations (typically >60 mEq/L), two CFTR mutations shown to be pathogenic, and/or multi-organ disease. By convention, sweat chloride levels in NCFBE are <60 mEq/L. NCFBE is therefore often considered a diagnosis of exclusion – if criteria are inadequate to establish a diagnosis of "cystic fibrosis," NCFBE may instead be entertained. Patients with NCFBE are not approved for Trikafta™, and do not have access to the drug. Based on a considerable body of evidence, we believe a large and surprising number of patients with NCFBE have a disease likely to exhibit significant clinical benefit from drugs such as Trikafta™ that activate CFTR-dependent ion transport, although such a notion has not been adequately tested or proposed previously.

In a subset of individuals with NCFBE, pathogenesis is likely due – at least in part – to CFTR deficiency, originating from inherited factors (i.e., asymptomatic CF carrier status, which occurs in approximately 1 of 30 Caucasians in the US and leads to ~50% decrease in CFTR mRNA and protein), acquired factors (e.g., due to chronic airway inflammation, infection, hypoxia, or toxin exposure), or a combination of these. For example, in patients with chronic airway infection and inflammation, or past toxin (e.g. nicotine) exposure, CFTR activity has been suggested to be negatively impacted – resulting in defects in anion secretion, airway surface liquid depletion and blunted mucociliary transport. Furthermore, CFTR mutation carriers, with an estimated half the level of functional CFTR compared to non-CFTR mutation carriers, are at increased risk for a range of adverse conditions, including NCFBE (Pignatti, Bombieri et al. 1995, Casals, De-Gracia et al. 2004, Miller, Comellas et al. 2020, Polgreen and Comellas 2022). Moreover, in a study of 122 individuals with NCFBE and normal sweat chloride concentrations, 22 (18%) were found to have one CFTR mutation and abnormal CFTR function in respiratory track epithelium that was intermediate between healthy controls and those with classical CF (Bienvenu et al. 2010).

Considerations regarding NCFBE

The incidence of NCFBE is increasing worldwide. Previously classified as a rare or orphan disease, the diagnosis has increased by 40% in the past several years (Flume, Chalmers et al. 2018) and the number of adults with bronchiectasis in the United States is estimated at 350,000 - 500,000 (Weycker, Hansen et al. 2017); i.e., much higher than the number of adults with CF (which amount to 30,000-40,000). Unfortunately, there are no curative therapies or medications specifically approved to address fundamental mechanisms that underlie NCFBE.

Trikafta™ is approved for patients with CF carrying at least one copy of the common F508del variant or a number of other CFTR abnormalities. Trikafta™ is a combination of three CF drugs, elexacaftor, ivacaftor, and tezacaftor (ETI), that helps CFTR proteins work more effectively. Patients with common forms of CF typically exhibit a robust pulmonary benefit from Trikafta™ within several days of initiating treatment (Middleton, Mall et al. 2019, Barry, Mall et al. 2021).

Trikafta™ is not prescribed or approved for NCFBE and has not been considered of benefit for patients with NCFBE. We hypothesize that a drug such as Trikafta™, that markedly activates both wild type and mutant CFTR *in vivo*, will enhance mucociliary clearance and should improve respiratory function (FEV_1) in a subset of patients with NCFBE. Even if one in ten patients with NCFBE shows significant improvement in FEV_1 following Trikafta™, this would constitute a major discovery and provide new hope for tens of thousands of individuals in the US with an otherwise untreatable and lethal lung disease.

Figure 1. Summary of trial design.

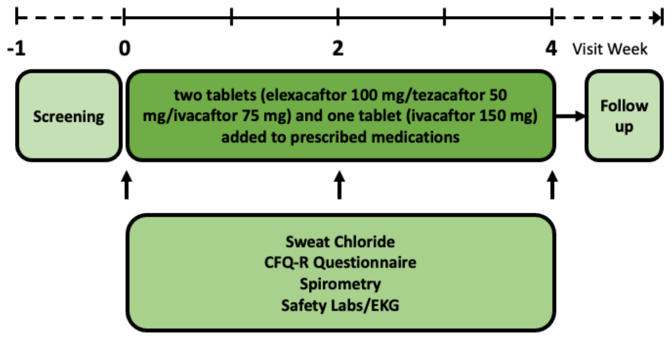

Trikafta™ treatment for an individual without known CFTR mutations

We recently treated a patient who had no known mutations in CFTR but exhibited clinical features of CF – including bronchiectasis and a modestly elevated sweat chloride – with Trikafta™. This patient experienced a robust improvement in lung function following 4 weeks of Trikafta™ therapy (Figure S1, Supplemental Materials). These results indicate that certain patients with severe bronchiectasis (and without diagnostic CFTR mutations) may nonetheless respond strongly to Trikafta™.

Emerging approaches to identifying patients with NCFBE most likely to exhibit a clinical response to Trikafta™

The nature and extent of *in vitro* model systems that predict pulmonary responsiveness to Trikafta™ are being evaluated by several laboratories, including our own. Reprogramming of adult somatic cells into iPS cells is a powerful approach that holds promise for regenerative and personalized medicine. This system presents potential advantages over classical *in vitro* cell-based and other models, and has been useful for generating efficient, high-performance tools for clinical translational research and drug development (Ebert, Liang et al. 2012). From a translational perspective, the use of patient-derived specimens may allow a comparative evaluation of drug efficacies *in vitro* to predict the therapeutic responses at an individual level, as recently shown by a number of investigators, including those participating in the current project (Crane, Kramer et al. 2015, Hawkins, Kramer et al. 2017, Jacob, Morley et al. 2017, Lopes-Pacheco 2019, Suzuki, Crane et al. 2020, Barillà, Suzuki et al. 2021, Brewington, Hong et al. 2021, Linnemann, Solomon et al. 2021).

<u>Summary</u> We believe NCFBE in many individuals will respond to stimulation of CFTR function in the airway, including patients with sweat chloride values that are normal or only mildly elevated (i.e., indicating diminished CFTR activity). The present study will comprise an open-label, one center trial of orally administered elexacaftor, tezacaftor and ivacaftor (Trikafta™) that will enroll

30 patients with NCFBE. Study subjects will have one known CFTR mutation and/or mildly elevated sweat chloride measurements (i.e., 30-60 mEq/L; Figure 1 summarizes trial endpoints). Patients with a diagnosis of CF will be excluded. A detailed schedule of events is provided under "Human Subject Involvement". The study will specifically and prospectively test iPS cells taken from patients with NCFBE to determine *in vitro* thresholds for predicting CFTR rescue *in vivo*. Using iPS cells differentiated to exhibit a respiratory epithelial phenotype, we will determine whether the cells can be used to predict FEV_1 response among individuals with NCFBE who receive Trikafta™.

4. Study Endpoints

- Primary Endpoints: iPSC-derived evidence of a Trikafta™ response and pulmonary function testing of FEV_1 (forced expiratory volume in one second) following Trikafta™ administration *in vivo*
- Secondary Endpoints: sweat chloride, NCFBE-directed quality of life tool, weight gain, safety laboratory evaluation/EKG

OBJECTIVES	ENDPOINTS	JUSTIFICATION FOR ENDPOINTS
Primary		
To determine whether iPS cells differentiated to airway epithelia -- and treated with ivacaftor/tezacaftor/elexacaftor *in vitro* -- can predict clinical responsiveness to Trikafta™ *in vivo*.	• Functional and biological correction of CFTR expressed in iPS cells as judged by short circuit current measurements in monolayers, and western blot analysis	• These *in vitro* tests represent standard functional and biochemical analyses that address CFTR rescue, with direct relevance to the *in vivo* situation.
To evaluate FEV_1 clinical responsiveness to Trikafta™ *in vivo*.	• Forced expiratory volume in one second (FEV_1).	• FEV_1 is a conventional endpoint and provides a direct measurement of patient health benefit among individuals with NCFBE.
Secondary		
To verify safety and further establish efficacy of Trikafta™ in enrolled patients.	• Sweat chloride test	• Provide supportive information regarding study intervention effect using *in vivo* measures

OBJECTIVES	ENDPOINTS	JUSTIFICATION FOR ENDPOINTS
	• Quality of life survey<br><br>• Standard laboratory tests and other measurements, as well as history and physical examination	known to indicate CFTR rescue<br><br>• A well-established tool for monitoring patient benefit<br><br>• Confirm safety of the FDA-approved drug

5. Study Intervention/Investigational Agent

Subjects will be given the Study Drug elexacaftor 100 mg/tezacaftor 50 mg/ivacaftor 75 mg (2 pills once daily in the morning) and ivacaftor (150 mg) once daily in the evening, as the FDA-registered agent, Trikafta™. Dose and schedule will be identical to what has already been FDA-approved for effective treatment of CF. The research pharmacy will be used since Trikafta™ is not approved for NCFBE.

The medicine will be dispensed by the hospital research pharmacy and delivered to the principal investigator or study team member. The principal investigator or health care providers will provide the medicine to the participant and answer any questions the participant might have. The study participants may also call the pharmacy if thay have questions about the medicine. The Investigational Drug Service (IDS) of the Research Pharmacy at Emory will distribute drug according to standard procedures for registered drugs of this type.

6. Procedures Involved

Human Subjects Involvement

**Procedures**	Screening/ Enrollment Day -28 to -1	Baseline Visit 1, Day 1	Study Visit 2 (phone call) Day 7 +/-2 days	Study Visit 3 Day 14 +/- 2 days	Study Visit 4 Day 28 +1/-2 days	Final Study Visit Day 56 +/-2 days
Informed consent	X					
Medical history	X	X	X	X	X	X
Physical exam	X	X		X	X	X
Cutaneous punch biopsy		X				X[C]
CFTR genotype	X					

**Procedures**	Screening/ Enrollment Day -28 to -1	Baseline Visit 1, Day 1	Study Visit 2 (phone call) Day 7 +/-2 days	Study Visit 3 Day 14 +/- 2 days	Study Visit 4 Day 28 +1/-2 days	Final Study Visit Day 56 +/-2 days
Spirometry	X	X		X	X	X
Oxygen saturation	X	X		X	X	X
Sweat chloride testing	X	X		X	X	X
Safety laboratory assessment[A]	X	X		X	X	X
Pregnancy Test[B]	X	X				X
EKG	X			X		
Drug accountability		X		X	X	
QOL Questionnaire	X	X		X	X	X
Administer Study Drug		X			X	
Concomitant medication	X	X	X	X	X	X
Adverse event assessment		X	X	X	X	X

[A] Renal and liver panels, CBC/Diff urinalysis
[B] Females of reproductive age
[C] If necessary to produce additional iPS cells

The study will examine iPSC-derived airway epithelial monolayers (an *in vitro* analytic tool) as a predictive means for identifying individuals with NCFBE most likely to show respiratory benefit from Trikafta™. As part of the protocol, we will perform an open-label clinical trial of orally-administered Trikafta™ in subjects with NCFBE. Subjects will be given the Study Drug elexacaftor 100 mg/tezacaftor 50 mg/ivacaftor 75 mg (2 pills once daily in the morning) and ivacaftor (150 mg) once daily in the evening, as the FDA-registered agent, Trikafta™. Dose and schedule will be identical to what has already been FDA-approved for effective treatment of CF. Trikafta™ is not approved for use in NCFBE. Enrollment is planned at Emory University. Patients enrolled in the trial will have one disease-causing CFTR variant and/or modestly elevated sweat chloride values (30-60 mEq/L), but without clinical findings sufficient for a diagnosis of cystic fibrosis.

Human subject participation in the 'main' study will consist of a screening day (-28), day 1 (+/-2), day 7 (+/- 2) [phone call visit], day 14(+/-2), day 28 (+1/- 2) and day 56 (+/-2) follow up (wash-out). The visits will include patient history with concomitant medication review, physical exam, safety laboratory assessments (renal and liver panels, CBC/Diff, urinalysis), serum and urine pregnancy testing, CFTR genotype, spirometry, O_2 saturation, sweat chloride test, cutaneous punch biopsy for iPSC derivation, EKG, drug accountability and questionnaire (QOL assessment), concomitant medication review, and adverse event assessment. No longer-term follow-up beyond day 56 is being planned (except for possible cutaneous biopsy by day 70 if needed (see below).

We anticipate the screening and enrollment visit will require 4-6 hours to complete the informed consent, medical history, O_2 saturation, concomitant medication review, physical exam, safety laboratory assessments (CBC/Diff, RENAL/LFT chemistry panel, and urinalysis),

EKG, CFTR genotyping, questionnaire, serum pregnancy test for females, sweat chloride test, and spirometry.

On day 1 (when subject returns), the visit will require approximately 6 hours to complete the concomitant medication review, adverse event assessment, interval medical history, physical exam, O_2 saturation, safety laboratory assessments (CBC/Diff, RENAL/LFT chemistry panel, and urinalysis), questionnaire, sweat chloride, urine pregnancy test for females, spirometry, cutaneous punch biopsy (3mm), drug dispensing, and drug administration. The cutaneous punch biopsy may occur at any time between the Screening Visit and Day 1 based on dermatology clinic schedule availability.

- <u>1 Cutaneous 3 mm punch biopsy (performed here (and on day 56 (or up to two weeks later) if necessary to produce additional iPS cells)):</u> In this procedure, a circular blade is rotated from epidermis to subcutaneous fat, yielding a ~ 3 mm cylindrical core of tissue. Extending the skin perpendicular to the incision line allows for facile closure, which can typically be accomplished with a single suture. Pain is well-managed by local subcutaneous anesthetic. A small fibrotic scar can be minimized by linear closure of the biopsy. Significant pain, bleeding, or infection are very rare.

The subject will complete a phone call visit on day 7 (+/-2) that will require approximately 30 minutes to complete concomitant medication review, interval medical history, and adverse event assessment.

On day 14 (+/-2) the subject will return for a visit that will require approximately 4-6 hours to complete concomitant medication review, interval medical history, adverse event assessment, physical exam, drug dispensing and drug return, questionnaire, safety laboratory assessments (CBC/Diff, RENAL/LFT chemistry panel, and urinalysis), sweat chloride test, EKG, O_2 saturation and spirometry.

On day 28 (+1/-2) we will conduct interval medical history, adverse event assessment, concomitant medications, physical exam, sweat chloride test, spirometry, O_2 saturation, safety laboratory assessments (CBC/Diff, RENAL/LFT chemistry panel, and urinalysis), questionnaire, and drug return. This visit should require 4-6 hours to complete. This visit should occur no later than Day 29 since a 28-day supply of Trikafta™ will be provided.

The 'wash-out' visit will be conducted on day 56 (+/-2) which will require approximately 4-6 hours to complete concomitant medication review, interval medical history, adverse event assessment, physical exam, safety laboratory assessments (CBC/Diff, RENAL/LFT chemistry panel, and urinalysis), questionnaire, sweat chloride, urine pregnancy test for females, cutaneous punch biopsy (3mm) if necessary to obtain additional cells, O_2 saturation, and spirometry. The cutaneous punch biopsy may occur within ± 14 days of the day 56 visit based on dermatology clinic schedule availability. No further data will be collected after all study procedures and measurements are completed.

Spirometry may be performed either pre- or post-bronchodilator at Screening. At all other visits, spirometry should be performed pre-bronchodilator. However, if patient is unable to withhold bronchodilator use at the Day 1 visit, then the spirometry assessment will be considered 'post-bronchodilator' and attempts should be made to collect spirometry post-bronchodilator at remaining visits.

In order for a spirometry assessment to be considered pre-bronchodilator, subjects should adhere to the following:

- Subjects should refrain from using short-acting bronchodilators (e.g., albuterol) or anticholinergic (e.g., ipratropium bromide [Atrovent®]) for more than 4 hours before the spirometry assessment.
- Subjects should refrain from using long-acting bronchodilator (e.g., salmeterol) for more than 12 hours before the spirometry assessment; and
- Subjects should refrain from using once-daily, long-acting bronchodilator (e.g., tiotropium bromide [Spiriva®]) for more than 24 hours before the spirometry assessment.

Sources of Patient-Derived Material:

- Review of patient medical records and demographic information
- History and physical exam
- Blood and urine for safety studies (<6 tablespoons of blood per visit)
- Sweat chloride results
- Questionnaire data
- Spirometry (FEV_1, FVC, and FEF25-75%) calculated per Hankinson equation
- Cutaneous punch biopsy (3 mm)/iPS cell derivation
- O_2 saturation
- EKG
- CFTR genotype
- Pregnancy test
- Drug accountability
- Adverse event assessment
- Concomitant medication review

Note: Data collection forms for the study are in preparation.

Subject recruitment plan

We plan to recruit 30 subjects with NCFBE into the 'main' study. We intend to include ~50% female and ~50% male subjects age ≥ 18. Subjects will be referred from clinicians participating in the trial (Emory University, Atlanta) or from outside institutions.

All individuals who consent to participate will be evaluated by clinicians at Emory Clinic. Travel, meals, accommodations, etc. for study visits will be covered by funds available through the

project, and a payment of $150 per visit (see also below) will be provided as a gift card to reimburse the subject for the time taken to conduct this study.

1. Informed Consent Process:

Informed consent will be obtained by well-trained study coordinators or investigators using an Institutional Review Board approved document. A consent to allow CFTR genotyping and sweat chloride will be offered as part of the 'lead in' study to identify potential study subjects with CFTR mutations or mildly elevated sweat chloride levels. A second consent form will be available for individuals found by the 'lead in' study to have a single disease-causing CFTR mutation and/or sweat chloride 30-60 mEq/L, but without clinical criteria necessary for a diagnosis of cystic fibrosis. Participants will be asked to sign the informed consent documents only after the study has been fully described to the subject and all questions have been answered. Participants will be offered at least 24 hours to review the consent before the research is to begin, although consent may be obtained on the day of screening if preferred by study subjects (e.g., those traveling from significant distance).

NCFBE is not associated with cognitive or other intellectual limitations. No vulnerable adult subjects are intended for enrolment in the trial. If, in the opinion of the study investigator, a prospective participant lacks sufficient understanding and is therefore not able to provide informed consent, that subject will not be enrolled. The ability to obtain consent represents criteria for inclusion in the trial.

2. Compensation for time and effort:

Travel, meals, accommodations, etc. for study visits will be covered by institutional or other funds (participant and caregiver companion). A reimbursement for time taken to complete the study of $150 per in-person visit and $30 at the Day 7 telephone call will be provided as a gift card. All payments will be pro-rated. A payment of $150 will also be provided if an unscheduled visit is needed (for multiple repeat assessments or cutaneous punch biopsy occurring on another day). If the unscheduled visit is needed just for a repeat laboratory sample collection, a payment of $30 will be provided. A payment of $150 will be provided for participation in the 'lead in' study.

7. Statistical Analysis Plan

Goals of the 'main' study are to determine the clinical response to Trikafta™ and whether *in vitro* responsiveness of iPS cell-derived airway monolayers predict Trikafta™ effectiveness. Primary outcomes comprise the ability of iPSC-derived monolayers to predict respiratory benefit from Trikafta™ after 4 weeks of *in vivo* treatment. Primary analysis will include an estimate of clinical response rate of Trikafta™ at week 4 of treatment and a 95% confidence interval using the exact method proposed by Blaker (Blaker, 2000). A responder is defined as any subject with an improvement, from baseline, in FEV_1 > 5% predicted. FEV_1 will also be considered continuously. A two-sided 0.05 alpha level paired t-test comparing the baseline and week 4 FEV_1 measurements, along with the test and 95% associated confidence interval, will be evaluated.

In this study, if at least 15% of subjects meet the definition of responder, we will view this as initial evidence of a favorable result. Overall response rate will be accompanied by an exact 95% confidence interval. To determine the role of iPS *in-vitro* response to Trikafta™, we will compare the Δ lsc (change in lsc) among Trikafta™ responders and Trikafta™ non-responders using an ANCOVA test. Additionally, we will correlate the Δ lsc obtained from *in vitro* experiments with the change in FEV_1% predicted (post minus pre) using Spearman's rank-order correlation with an associated 95% confidence interval. We will also test whether the iPS response reaches a CFTR functional threshold of 30% wild-type levels (a magnitude of activity in primary airway epithelial cells viewed as potentially relevant to clinical benefit). In a sensitivity analysis, we will replicate the FEV_1 analysis with sweat chloride responses and determine the *in vivo* response rate.

Secondarily, we will serially evaluate clinical outcomes of interest prior to and after the start of therapy. Change in clinical measurements (in addition to spirometry – sweat chloride, weight/BMI, quality of life) will be monitored. A complementary analysis evaluating the percentage of participants with ≥15 mmol/L decline in sweat chloride will also be reported. With a previously estimated 9.7 mmol/L standard deviation of the change in sweat chloride for successful modulator trials (Boyle et al., 2014; Vermeulen et al., 2017), an anticipated 6% of responders would be expected by chance alone. Therefore, the treatment response prevalence will be compared to a reference rate of 6%. For longitudinal FEV_1 through day 28 and for secondary endpoints (absolute change in sweat chloride, QOL questionnaire, and weight/BMI from baseline through day 28), means at each time point and changes over time will be summarized along with corresponding confidence intervals estimated by mixed effects models to account for within-subject correlation.

Changes in FEV_1 following treatment with Trikafta™ will be correlated with secondary clinical outcome measures. A non-linear Spearman's correlation coefficient will be estimated for each measure paired with iPS cell responses *in vitro*, with corresponding fitted curves displayed graphically along with coordinates for participant's paired *in vivo* and *in vitro* responses.

For other outcomes of interest including sweat chloride levels, quality of life, and weight/BMI, we will also apply a mixed effect model to examine the change in these measures over the study duration. Models will include a subject-specific intercept, to account for variability among subjects prior to the start of the treatment, and a categorical variable for time (baseline, day 14, day 28, day 56). A Dunnet's post-hoc comparisons procedure will be used to determine which time points demonstrated a significant change from baseline. Using the same mixed effects models, we will include Δ Isc as predictors of *in vivo* improvement. We will also test the interaction between time and Δ lsc to determine when *in vitro* response is most associated with change in clinical response. Adverse events and other safety endpoints will be tabulated and reported using counts and percentages or means and standard deviations, as appropriate.

Because clinical parameters may vary among patients prior to the start of therapy, we will also approach the analysis using a modified N of 1 design configured for the purpose of comparing iPS studies in contrast to the aggregate analysis described above. Using the framework of single

subject research, we will evaluate therapeutic response in individual subjects by an interrupted time series (ITS) method. Briefly, in the absence of an independent control group, ITS analysis is a quasi-experimental design in which each subject acts as their own control and is followed serially in time prior to and after an interruption (in this case, the start of treatment). If the treatment has causal impact, the post-intervention time series will have a different level or slope than the pre-intervention series. Time series models also have the power to test and correct for possible cyclical patterns and outliers. Using segmented regression analysis, we will conduct ITS models for each of the subjects enrolled in the trial. A significant change in slope from the pre-treatment period will indicate a significant treatment effect. ITS models will be constructed for each outcome of interest where historical and pre-treatment data are available. The change in slope will also be correlated with the *in-vitro* response using Spearman's rank-order correlation coefficient.

All analyses will be conducted using the intention to treat principal, where all subjects will be included in analysis regardless of how long they took the study medication. Statistical significance will be assessed at the 0.05 level and analyses conducted using SAS v. 9.4 (SAS Institute; Cary, NC).

Sample size

We will test the null hypothesis that the true Trikafta™ response rate of NCFBE individuals is ≤ 1% vs. the alternative hypothesis that the true Trikafta™ response rate is ≥ 15%. We will initially enroll 16 subjects. If none of these subjects exhibit a clinical response (defined as > 5% improvement of % predicted FEV_1 from baseline), the study may be considered for termination. Otherwise, we intend to enroll a total sample size of 29 individuals (or 30 subjects, in the unlikely event that an individual discontinues participation; i.e., subjects who do not complete the study will be replaced). In this design, if Trikafta™ is ineffective, the probability of early termination after 16 subjects is 85% and only a 2.8% chance of concluding the drug is effective (type I error). If Trikafta™ is effective, there is a 90.04% chance of reaching this conclusion. If fewer than 2 patients (of 29 patients total) of the overall cohort show a clinical effect, Trikafta™ will be considered ineffective. To demonstrate the ability of iPS cells to predict clinical benefit, 16 (29) patients will provide at least 80% power to detect a significant Spearman correlation of at least r = 0.8 (.53) between the changes in clinical outcomes with changes in short circuit current as measured in iPS cells. Tabular details of this analysis are provided below. Power calculations were performed using PASS v. 14 (Kaysville, UT) with a two-sided Z-test and a significance level of 0.05.

	**Cumulative # of responses**	**Decision**	**95% Confidence Interval (CI)**
**Stage 1: Enter 16 subjects**	0	Consider Termination of the trial because the agent is ineffective.<br>**Response rate is ≤1%**	LB of 95% CI is < 1%
	At least 1	Inconclusive result, continue	95% CI contains 1%

		trial (proceed to stage 2).	
**Stage 2: Enter 14 additional subjects**	1 or less (stage I + stage II)	Consider that the agent is ineffective. Response rate is ≤1%	LB of 95% CI is less than 1%
	2 or more (stage I + stage II)	Drug may be effective Response rate is greater than 1% and not different from 15%	95% CI contains 15% and LB is > 1%
	6 or more (stage I + stage II)	Drug is effective Response rate is at least 15%	95% CI LB ≥ 15%
LB = Lower Bound, UB = Upper Bound<br>Note: Thirty total patients will be enrolled in the unlikely event that a subject discontinues participation (e.g., due to elevated liver function tests, skin rash, or for other reasons).			

With a sample size of 16 (29) participants, we anticipate a 97% (>99%) power to detect a mean sweat chloride decrease of 10 mmol/L assuming a standard deviation of 9.7 mmol/L (Boyle, Bell et al. 2014, Vermeulen, Le Camus et al. 2017) under a two-sided 0.05 alpha-level paired t-test. With a higher standard deviation of 15 mmol/L, the power decreases to 70.3% (93.4%) to detect a mean decrease of 10, but we retain 96.2% (>99%) power to detect the hypothesized mean decrease of 15 mmol/L with a sample size of 16 (29) patients.

SAEs and AEs will be tabulated using standard coding terms sorted by System Organ Class (SOC). The incidence of AEs will be tabulated by seriousness and severity. The number of SAEs and AEs will be summarized as follows: (i) The proportion of subjects with at least one (S)AE, (ii) The average number of (S)AEs per subject, and (iii) The rate of (S)AEs per subject week of follow-up.

Expected Outcomes Subjects will be recruited by study investigators as above. Please note that based on considerable past experience conducting clinical trials and past collaborations with NCFBE clinical leadership throughout the US, we do not have concerns regarding our ability to perform the patient-oriented study described here, or to propagate and characterize iPS cells (Crane, Kramer et al. 2015, Mou, Brazauskas et al. 2015, Hawkins, Kramer et al. 2017, Jacob, Morley et al. 2017). The 'main' study protocol will evaluate the clinical benefit of Trikafta™ in patients with NCFBE. In addition, the study will furnish one test of our hypothesis that thresholds of CFTR activity in differentiated iPS cells *in vitro* predict benefit following Trikafta™ *in vivo*. If a strong correlation can be established between Trikafta™ response *in vitro* and *in vivo* (e.g., FEV_1), the clinical results will provide evidence regarding usefulness of *in vitro* models for predicting clinical efficacy in this setting.

Scientific Rigor All *in vitro* and *in vivo* studies will be conducted with appropriate "n" so that therapeutically relevant conclusions can be drawn. Multiple biologic or technical replicates will be performed and evaluated in blinded fashion by an experienced statistician. NCFBE is represented in both sexes, and we expect equal enrollment of male and female individuals.

8. Data and/or Specimen Banking

- Patient skin biopsies and iPSCs derived from skin samples will be anonymized (identifiable only by a patient-specific identification number). The frozen iPSCs will be maintained in liquid nitrogen at the University of Texas, Houston by collaborating laboratories for two years after completion of the study, and then destroyed. Investigators conducting laboratory tests of iPSCs and primary airway epithelium will have access to specimens but will not have access to patient names or identifiers. This approach will be approved by the University of Texas at Houston IRB.
- Data for each subject (Section 6) will be maintained in patient-specific study folders in anonymized fashion (as above) and stored in a locked and secure cabinet in a limited access study room. Patient data maintained as computer files will be password protected and maintained on a limited access, user specific network drive. All patient data specific to the study will be kept for two years after trial completion and then destroyed.
- Links between patient identification numbers and study subject data will be kept in a secure password protected computer file by project leadership (Sorscher and Hunt) and made available to other study personnel as deemed necessary.
- Participant confidentiality and privacy will be strictly held in trust by the participating investigators, their staff, and others responsible for the intended interventions. This confidentiality is extended to cover testing of biological samples and other tests in addition to the clinical information relating to participants. Therefore, the study protocol, documentation, data, and all other information generated will be held in strict confidence. No confidential information concerning the study, or the data will be released to any unauthorized third party without prior written approval. Study personnel will have access to sources of information regarding participants as delineated by the informed consent document signed by all study subjects.
- All research activities will be conducted in as private a setting as possible.
- The clinical investigators, other authorized representatives, representatives of the Institutional Review Board (IRB), or regulatory agencies may inspect all documents and records required to be maintained by the investigators, including but not limited to, medical records (office, clinic, or hospital) and pharmacy records for the participants in this study. The clinical study site will permit access to such records.
- The study participant's contact information will be securely stored at each clinical site for internal use during the study. At the end of the study, all records will continue to be kept in a secure location for as long a period as dictated by the reviewing IRB, Institutional policies, or other requirements (currently planned for two years post-study completion).
- Study participant research data, which is for purposes of statistical analysis and scientific reporting, will be transmitted and stored securely as above. This will not include the participant's contact or identifying information. Rather, individual participants and their research data will be identified by a unique study identification number. The study data entry and study management systems used by clinical research staff will be secured and

password protected. At the end of the study, all study databases will be de-identified and archived by the PI. With regard to other patient related aspects of confidentiality, all interviews and procedures will be held in a private room.

9. Sharing of Results with Participants

Sharing of information with study participants and others Subjects enrolled in the study will have access to their study information once participation in the trial is complete. In addition, study data may be provided to the patient's pulmonary (or other) physician, if desired by the study subject. This is important, since patients enrolled in our trial who exhibit a favorable response to one month of Trikafta™ treatment are likely to benefit from continued therapy. Because the drug is expensive (~ $300,000/year) and not "on label" for NCFBE, third-party payers may not reimburse for Trikafta™ treatment. Patients and their physicians, however, can sometimes use data as provided in our protocol to gain third-party approval for Trikafta™ reimbursement, indicating the importance of making participant data available as intended here.

Reporting of Study Results

De-identified data from this study, including de-identified genetic information, may be shared with the research community at large to advance science and health. Any personal information that could identify an individual study subject will be removed or coded before that information is shared with the research community. This will ensure that, by current scientific standards and known methods, it is extremely unlikely that anyone would be able to identify a study subject from the information we share. Despite these measures, it is not possible to guarantee anonymity of personal data.

Reporting in Medical Records

If a study participant does not have an Emory medical record, one will be established since Emory providers or facilities are furnishing services and procedures for this trial. Copies of the consent form/HIPAA authorization will be placed in the Emory medical record. Emory may generate study information about the participants that can assist with care. For example, results of 'main' study tests or procedures (spirometry) may help with management of certain study subjects. Clinical trial results may therefore be placed in the Emory medical record. Anyone with access to the medical records will therefore also have access to all relevant study information. The confidentiality of the study information in the medical record will be protected by measures such as HIPAA, although state and federal laws may not protect the research information from disclosure.

The results of some study tests and procedures will be used only for research purposes and will *not* be placed in the medical record. For example, such items include: information resulting from the skin punch biopsy used to test *in vitro* cell response to Trikafta™. *In vitro* CFTR testing and

procedures done in the research laboratory (genotyping or sweat testing for the 'lead-in' study) or at non-Emory locations may not become part of the Emory medical record. If a participant decides to be in this study, it is up to the participant to inform health providers at other institutions.

Types of findings that may arise in this study (anticipated or otherwise) are summarized under Section 16 (Risks to Participants). All clinically significant findings will be reported to the study Data Monitoring Committee (DMC) and IRB as part of semi-annual, annual, or more expedited reporting according to DMC charter and IRB guidelines. A copy of the DMC charter is attached to this submission.

Unanticipated or incidental findings observed during the study that impact safety, patient risks, efficacy, and likelihood of enrolling or continuing of individual patients in the study will be communicated to trial participants. A statement in the informed consent reads as follows:

> "It is possible that the researchers will learn something new during the study about the risks of being in it. If this happens, they will tell you about it. Then you can decide if you want to continue to be in this study or not. You may be asked to sign a new consent form that includes the new information if you decide to stay in the study."

The consent also notes:

In Case of Injury

If you get ill or injured from being in the study, or if new findings result from the study that require additional medical care, Emory will help you get medical treatment. Emory and the study supporter have not, however, set aside any money to pay you or to pay for this medical treatment. The only exception is if it is proven that your injury or illness is directly caused by the negligence of an Emory or study supporter employee. "Negligence" is the failure to follow a standard duty of care.

If you become ill or injured from being in this study, your insurer will be billed for your treatment costs. If you do not have insurance, or if your insurer does not pay, then you will have to pay these costs.

If you believe you have become ill or injured from this research, you should contact Dr. Sorscher at telephone number XXX-XXX-XXXX or Dr. Hunt at XXX-XXX-XXXX. *You should also let any health care provider who treats you know that you are in a research study.*

Costs

There will be no costs to you for participating in this study, other than basic expenses like transportation, which will be reimbursed after each visit. You will not be charged for any of the research activities. If the study procedures result in any medical complications that would not fall under "injury" as discussed above, the cost of treatment for those complications may be charged to you or your insurance.

10. Study Timelines

From screening to the last follow-up visit, individual participant's participation in the study will be approximatley 4 months.

Anticipated enrollment of all study participants will require 2 years.

The estimated date for the investigators to complete this study is August 31, 2025.

11. Inclusion and Exclusion Criteria

In order to gain information regarding numbers of individuals with NCFBE who may be eligible for our trial, patients at Emory followed with a diagnosis of bronchiectasis (who do not have clinical criteria sufficient for a diagnosis of cystic fibrosis) will be asked to consider participating in a 'lead in' study that will determine the subject's CFTR genotype and sweat chloride. A separate consent form will be utilized for the 'lead in' study. We anticipate approximately 300 subjects will participate in the 'lead-in' study.

Subjects who exhibit a single CF-causing mutation in CFTR (e.g., F508del) and/or sweat chloride 30-60 mEq/L will be approached about their interest in reviewing the consent form for the 'main' study ("iPSC derivation and *in vivo* Trikafta™ treatment for 4 weeks").

Details regarding the 'main' study

We anticipate the screening and enrollment visit for the 'main' study will require 4-6 hours to complete the informed consent, medical history, O_2 saturation, concomitant medication review, physical exam, safety laboratory assessments (CBC/Diff, RENAL/LFT chemistry panel, and urinalysis), EKG, CFTR genotyping, questionnaire, serum pregnancy test for females, sweat chloride test, and spirometry.

Inclusion Criteria:

- Provision of signed and dated informed consent form
- Stated willingness to comply with all study procedures and availability for the duration of the study
- Male or Female age ≥18
- Radiologic and other clinical evidence leading to a diagnosis of NCFBE
- 1 CF-causing mutation and/or sweat chloride measurement ≥ 30 mEq/L and ≤ 60 mEq/L
- Able to perform spirometry meeting ATS criteria for acceptability and repeatability, and FEV_1 40-90 mEq/L
- Clinically stable in the past 4 weeks with no evidence of bronchiectasis exacerbation (prior to Screening AND Day 1)
- Willingness to use at least one form of acceptable birth control including abstinence or condom with spermicide. This will include birth control for at least one month prior to screening and agreement to use such a method during study participation for an additional four weeks after the last administration of Study Drug

- Ability to take Trikafta™
- Agreement to adhere to all current medical therapies as designated by the study physician

Exclusion Criteria

- Diagnosis of cystic fibrosis
- Documented history of drug or alcohol abuse within the last year
- Pulmonary exacerbation or changes in therapy for pulmonary disease in the 4 weeks prior to screening
- Listed for lung or liver transplant at the time of screening
- Cirrhosis or elevated liver transaminases > 3X ULN
- Pregnant or breastfeeding
- Inhibitors or inducers of CYP3A4, including certain herbal medications and grapefruit/grapefruit juice, or other medicines known to negatively influence Trikafta™ administration
- History of solid organ transplant
- Active therapy for non-tuberculosis mycobacterial infection or any plan to initiate non-tuberculosis mycobacterial therapies during the study period
- Known allergy to Trikafta™
- Treatment in the last 6 months with an approved CFTR modulator
- Any other condition that in the opinion of the lead investigators might confound results of the study or pose an additional risk from administering Study Drug
- Treatment with another investigational drug or other intervention within one month prior to enrollment, throughout the duration of study participation, and for an additional four weeks following final drug administration.
- Evidence of cataract/lens opacity determined to be clinically significant by an ophthalmologist at or within 3 months prior to the Screening Visit

12. Population

The following populations will be excluded from participation in this study:

- Adults unable to consent
- Individuals who are not yet adults (infants, children, teenagers)
- Pregnant women
- Prisoners
- Cognitively impaired or Individuals with Impaired Decision-Making Capacity
- Individuals who are not able to clearly understand English

There are no exclusions based on race or ethnicity. Further, we are not utilizing racial and ethnic classification of subjects for descriptive statistics, within an explanatory model, or as a variable to explain differences between patients.

13. Local Number of Participants

We anticipate that the 'lead-in' study (to gain information regarding numbers of individuals with NCFBE who may be eligible for the drug treatment ('main' trial) will primarily include patients at Emory followed with a diagnosis of bronchiectasis (who do not have clinical criteria sufficient for a diagnosis of cystic fibrosis). Approximately 300 participants will be accrued locally into the 'lead-in' study.

Subjects who exhibit a single CF-causing mutation in CFTR and/or sweat chloride 30-60 mEq/L will be approached about their interest in reviewing the consent form for the 'main' study (i.e., involving iPSC derivation and *in vivo* Trikafta™ treatment for 4 weeks). We will enroll 30 subjects accrued locally or via physician referral.

For the 'main' study, we plan to recruit 30 subjects with NCFBE. We intend to include ~50% female and ~50% male subjects age ≥ 18. Subjects will be referred from clinicians participating in the trial (Emory University, Atlanta), or from outside institutions. We anticipate that the study population will be 50% male, but >80% white, since most individuals who are carriers of a CFTR mutation are Caucasian. However, efforts will be made to enroll study subjects from non-Caucasian populations who are eligible (e.g., by conducting full exome CFTR analysis to identify rare CFTR mutations found more frequently among non-white individuals, and utilizing mildly elevated sweat chloride as an entry criterion (i.e., in addition to genotype)). Findings from the 'lead-in' study will be compiled in anonymous fashion and using patient identification numbers as described in Section 8. Because CFTR genotypes and sweat chloride for the 'lead-in' study will typically be conducted in a non-CLIA research laboratory, this screening data will not be included in the patient medical record. For subjects who enroll in the 'main' study, report of genotype and CLIA approved sweat testing will be performed and results entered in the medical record.

14. Recruitment Methods

In order to gain information regarding numbers of individuals with NCFBE who may be eligible for our trial, patients at Emory followed with a diagnosis of bronchiectasis (who do not have clinical criteria sufficient for a diagnosis of cystic fibrosis) will be asked to consider participating in a 'lead in' study that will determine the subject's CFTR genotype and sweat chloride. A separate consent form will be utilized for the 'lead in' study. Subjects who exhibit a single CF-causing mutation in CFTR and/or sweat chloride 30-60 mEq/L will be approached about their interest in reviewing the consent form for the 'main' study ("iPSC derivation and *in vivo* Trikafta™ treatment for 4 weeks")

We have currently identified 7-8 potential subjects for the 'main' study based on known clinical findings among these individuals. Data from the 'lead-in' study (CFTR mutation analysis – predeominantly for the common F508del variant and sweat chloride level) will typically be conducted in a research laboratory (non-CLIA approved), and will not be included in the patient record.

Subjects will be referred from clinicians participating in the trial (Emory University, Atlanta), or from outside institutions. We estimate up to 30% of subjects may be referred from outside institutions, primarily involving individuals from the Atlanta area.

15. Withdrawal of Participants

Criteria for discontinuing the 'main' study intervention (e.g., halting rules), may include any monitoring test(s) and associated clinical decision point(s) which in the opinion of the clinical investigators, qualified designees, or Data Monitoring Committee are viewed as placing the patient at significant risk if Trikafta™ administration is to continue. This could include, for example (but is not limited to) worsening liver function or abnormalities associated with Trikafta™ administration (see also below). Discontinuation from Trikafta™ does not mean discontinuation from the 'main' study, and remaining study procedures should be completed as indicated by the study protocol and lead investigators. If a clinically significant finding is identified (including, but not limited to changes from baseline) after enrollment, the investigators or qualified designees, and/or Data Monitoring Committee (also referred to as Data Safety Monitoring Committee, DSMC) will determine whether any change in participant management is needed. Any new clinically relevant adverse finding will be reported as an adverse event (AE).

The data to be collected at the time of 'main' study intervention discontinuation will include:

- All measurements described for the day 56 follow-up/washout visit

Participants are free to withdraw from participation at any time upon request.

An investigator may discontinue or withdraw a participant from the study for the following reasons:

- If any clinical adverse event, laboratory abnormality, or other medical condition or situation occurs such that continued participation in the study would not be in the best interest of the participant
- Disease progression which requires discontinuation of the 'main' study intervention
- If the participant meets an exclusion criterion (either newly developed or not previously recognized) that precludes further study participation
- Participant is unable to receive Trikafta™ for 3 days
- Pregnancy
- Significant study intervention non-compliance

The reason for participant discontinuation or withdrawal from the 'main' study will be recorded. An attempt will be made to replace subjects who sign the informed consent form and receive Trikafta™, and subsequently withdraw, or are withdrawn or discontinued from the study.

A participant will be considered lost to follow-up if he or she fails to return for any scheduled visit and is unable to be contacted by the ‘main’ study site staff.

The following actions will be taken if a participant fails to return to the clinic for a required ‘main’ study visit:

- The study investigators will attempt to contact the participant and reschedule the missed visit within 2-5 days, and counsel the participant on the importance of maintaining the assigned visit schedule and ascertain if the participant wishes to and/or should continue in the ‘main’ study.
- Before a participant is deemed lost to follow-up, the investigator or designee will make every effort to regain contact with the participant (where possible, 3 telephone calls and, if necessary, a certified letter to the participant’s last known mailing address or local equivalent methods). These contact attempts should be documented in the participant’s medical record or study file.
- Should the participant continue to be unreachable, he or she will be considered to have withdrawn from the ‘main’ study with a primary reason of lost to follow-up.

16. Risk to Participants

Blood Draws
Possible risks include hematoma at the needle site and minimal pain due to the venous puncture procedure.

Sweat chloride testing
The Macroduct system is used in hospitals and clinics worldwide to perform sweat chloride testing. This is considered a very safe, painless procedure, but rare minor burns have been reported at the electrode site (although participants may show no discomfort during the test). To further minimize risk, all equipment will be properly cleaned and evaluated before and after the procedure by personnel trained in performance of this assay.

Risks of the punch biopsy

Significant pain, bleeding, or infection are very rare with this type of punch biopsy. Investigators may use a topical anesthetic on the skin in order to minimize pain, although study subjects might feel minor discomfort (like a scratch) when the topical anesthetic is administered, and the next day. There is the possibility of a very small scar.

Risks from Study Drug (Trikafta™ (elexacaftor 100 mg/tezacaftor 50 mg/ivacaftor 75 mg (2 pills once daily in the morning) and ivacaftor (150 mg) once daily in the evening)

There may be side effects from the Study Drug or procedures that are not known at this time. Examples of adverse reactions known in patients receiving Trikafta™ are listed below.

The most common (occurring 5% or more) risks and discomforts expected in this study are:

Headache	17%
Upper respiratory tract infection	16%
Abdominal pain	14%
Diarrhea	13%
Rash	10%
ALT increased (a liver enzyme)	10%
Nasal congestion	9%
Blood CPK increased (a muscle enzyme)	9%
Rhinorrhea (runny nose)	8%
Rhinitis (nose irritation)	7%
AST increased (a liver enzyme)	9%
Influenza	7%
Sinusitis	5%
Blood bilirubin increased (a liver enzyme)	5%

Increased liver enzymes (ALT or AST) in the blood have been observed in some subjects. Very high levels of these enzymes could lead to stopping of Study Drug. The abnormal blood tests may improve after Study Drug is stopped. In some severe cases, high liver enzymes can become permanent or even life-threatening.

<u>Possible Risks Based on Animal Studies</u>
In a study in which ivacaftor (a component of Trikafta™) was given to newborn rats, cataracts (cloudiness of the lens of the eye) were seen. No cataracts were seen in studies of older animals (rats and dogs) dosed with ivacaftor for longer periods of time. The importance of this finding in humans is unknown.

<u>Drug Interaction Risks</u>
The combination of the Study Drug and other medications, dietary supplements, natural remedies, and vitamins could be harmful to trial participants. Subjects will be asked to inform study staff about every medicine, dietary supplement, natural remedy, and vitamin (or change in medicine) while they are in the study. The study staff will review all medications (including herbal medications, such as St. John's Wort, grapefruit or grapefruit juice) that should not be utilized during the study because herbal compounds such as these can alter metabolism of Trikafta™.

<u>Risks of Discontinuing Study Medication:</u>
Once the four-week treatment period is complete, participants will no longer receive Trikafta™. There is a chance that the NCFBE will improve when taking Trikafta™. However, NCFBE symptoms may appear to worsen after the participant stops taking Trikafta™ as respiratory function returns to baseline.

<u>Women:</u>

To protect against possible side effects of the Study Drug, women who are pregnant or nursing a child may not take part in this study. If a participant becomes pregnant, there may be risks to the participant, the embryo, or fetus. These risks are not yet known. If the participant is a woman of childbearing ability, the participant and the Study Doctor must agree on a method of birth control to use throughout the study. Pregnant women will be taken out of the study.

Possible risks to non-study participants:
The effect of the Study Drug on sperm is not known. To protect against possible side effects, if the female partner of a male participant becomes pregnant during the Study, they should notify the Study Doctor right away. If a male participant's female partner becomes pregnant, he will need to stop Study Drug immediately.

Study Drug should be kept out of the reach of children or anyone else who may not be able to read or understand the label. Participants should not allow anyone else take the Study Drug.

It is possible that new information regarding Study Drug or participation in the trial during the course of the clinical protocol will become evident. If this happens, patients will be informed so that they can make a decision about continuing in the study (see also Section 9). Participants in the trial may be asked to sign a new consent form that includes the new information in order to continue in the study.

17. Potential Benefits to Participants

This study is not designed to benefit the participant directly. NCFBE may improve in this study but it may not, and it may also become worse. This study will provide new information regarding whether cells from the skin can be used to predict patients with NCFBE who respond best to new drug therapies.

18. Compensation to Participants

Travel, meals, accommodations, etc. for study visits will be paid for by the study and an incentive payment of $150 will be provided at Screening, Day 1, Day 14, Day 28 and Day 56. An incentive payment of $30 will be provided at the Day 7 telephone contact. For adult subjects who are accompanied by a caregiver, travel expenses, meals, and accommodations will also be provided for the caregiver. If an unscheduled visit is needed, an incentive payment of $150 will be provided. An unscheduled visit may occur if multiple procedures need to be repeated or if the punch biopsy needs to be performed on a day other than a scheduled visit. An incentive payment of $30 will be provided if the unscheduled visit is only for a repeat blood draw. All payments to study subjects will be pro-rated.

Participants will be asked to fill out a tax form, including Social Security or Taxpayer Identification Number, in order to be reimbursed, depending on the amount and method of payment. Some payments may be mailed to the subjects house, which may be seen by others in the household. Participants can decline payment if they are concerned about confidentiality, or they can talk to the study team to see if there are other payment options.

19. Data Management and Confidentiality

All data and bankED biospecimens (iPSCs derived from skin biopsies) in this study will be de-identified, assigned a patient code identification number, and maintained in a secure location. Hard copies of data will be kept in a locked cabinet within a limited access study room and/or in password protected computer files. De-identified skin biopsies will be transported by overnight carrier from Emory to University of Texas at Houston. iPSCs will be stored in liquid nitrogen (a secure and key-accessible room) at the University of Texas at Houston. Only certified clinical coordinators and healthcare professionals who have completed institutionally assigned clinical trial accreditation will receive, transport, and transmit data and biospecimens, and have access to study materials of this type. Patient codes/identifiers will only be available to study leadership (Hunt, Sorscher), with other accredited and IRB-approved clinical team members provided access if needed. Data verification and quality control will be conducted with care and using standard methodology. Data and biospecimens will be maintained under secure storage for a period of two years after completion of the study. If a participant declines to participate in all portions of the study, that individual will not be assigned a study ID number and the study coordinators will refrain from collecting any data on the participant. If the individual agrees to participate in some portions of the study but not others, that individual will be assigned a study ID number and the study coordinator will be instructed to collect data only on those aspects of the study to which the subject has agreed to participate. These procedures will help prevent unauthorized inclusion of patient data in the database.

20. Plans to Monitor the Data to Ensure Safety of Participants and Data Integrity

Select one of the following (do not delete this table; review the guidance document for definitions):	
☐ Medium Complexity	
☐ High Complexity Category A	
☒ High Complexity Category B<br>*If choosing this category for a study under an IND or IDE because you believe the study intervention does not significantly impact morbidity or mortality, please provide your rationale:*	

Data Safety Monitoring Plan

Oversight for this 'main' study will be performed by a Data Monitoring Committee (DMC) Chair. The DMC (also called a Data Safety Monitoring Committee, DSMC) will be selected in consultation with the Chair to specifically oversee the clinical study. The DMC will consist of at least two physicians experienced in treating patients with lung diseases, CF, and/or NCFBE, a DMC coordinator and a biostatistician. The DMC Chair will convene a protocol conference call with committee members so that the DMC can approve the monitoring plan and review any concerns regarding the current protocol prior to initiation of study enrollment.

The DMC is primarily responsible for reviewing safety data which will be provided on a continual basis throughout the performance of the study with semiannual meetings, as well as reviewing an interim analysis report after 16 patients have completed the 56-day protocol. The DMC may recommend early termination or modification of the trial for reasons of study subject safety. Subjects will be monitored for adverse events (AEs) and serious adverse experiences (SAEs) by the Investigators, the Institutional Review Board, and the DMC. The DMC will also provide recommendations needed to assist the investigative team.

Study Monitoring: The Emory University self-monitoring tool will be customized to fit the needs of the current study. This tool will be utilized by the study team for monitoring progress of the trial and assuring data accuracy and protocol compliance. Study monitoring will take place at least once a year.

Clinical site monitoring is conducted to ensure that the rights and well-being of trial participants are protected, that the reported trial data are accurate, complete, and verifiable, and that the conduct of the trial is in compliance with the currently approved protocol/amendment(s), with International Conference on Harmonization Good Clinical Practice (ICH GCP), and with applicable regulatory requirement(s). The self-monitoring process and study team will visually inspect study records collected since each previous monitoring exercise. The scope of the monitoring shall include the informed consent process, eligibility, CRFs, biological specimen tracking and AE reporting (see also Table 3, below).

Quality control (QC) procedures will be implemented beginning with the data entry system. Data QC checks that will be run on the database will be generated. Any missing data or data anomalies will be communicated for clarification/resolution.

A designated member of the study team will provide the completed self-monitoring tool to the study PIs and team. The study PIs will document receipt and review of the monitoring report, resolutions and/or corrective actions to findings on a Site Monitoring Log. The monitoring reports will be shared with the Emory Clinical Trials Audit and Compliance (CTAC) office, and with the DMC. Protocol deviations/violations will be reported to the Emory IRB per reporting guidelines and will be reviewed by the DMC in a timely fashion or during regular meetings, or more frequently as needed.

This study may be temporarily suspended or prematurely terminated if there is sufficient reasonable cause. Written notification, documenting the reason for study suspension or termination, will be provided by the suspending or terminating party to study participants, investigators, and any other regulatory authorities. If the study is prematurely terminated or suspended, the Principal Investigator (PI) will promptly inform study participants, the Institutional Review Board (IRB), and will provide the reason(s) for the termination or suspension. Study participants will be contacted, as applicable, and be informed of changes to study visit schedule.

Circumstances that may warrant termination or suspension include, but are not limited to:
- Determination of unexpected, significant, or unacceptable risk to participants
- Insufficient compliance to protocol requirements
- Data that are not sufficiently complete and/or evaluable
- Determination that the primary endpoint has been met
- Determination of futility

Individual subject stopping rules are described under Section 15, “Withdrawal of Participants.”

In summary, one goal of the present study is to evaluate clinical benefit of a CFTR modulator in patients with NCFBE. In this protocol, the highest level of stringency will be applied. This will include: 1) DMC, as described above, 2) detailed interim evaluation, 3) generation of case report forms for all study subjects, 4) statistical analysis, tabular summaries, and leading-edge evaluation to test clinical benefit, 5) compiling data in SAS or other well-accepted formatting, and 6) timely publication of data, etc. Logistical support and resources of the type intended here are provided by the Institution for physician-orchestrated clinical trials at Emory. Our intent will be to furnish a clinical profile that provides high stringency and rigor. We recognize that for the present clinical trial, data will not be used for submission to FDA or towards label expansion of Trikafta™. However, by providing a preliminary data set and offering this information early and openly, we will advance new information that will help guide more extensive studies of NCFBE, Trikafta™, and iPSC cell analysis among other patients with the disease.

21. Monitoring Table 3

Please address the specific details below. Please do not alter the table and leave all template text to assist in a quick review. If deemed not applicable, please provide rationale. Note that the identified monitor(s) should be listed on the study’s delegation of authority log, if not monitored by a CRO.

DSMP Requirement	How this Requirement is Met	Frequency	Responsible Party(ies)
Site Monitoring at pre-determined intervals: The Principal Investigator has a responsibility to	*There should be a standard operating procedure to review data (whether a sample or 100%) at pre-determined*	*At a minimum, a review is required annually when no one has been*	*Delegate a responsible party for each requirement below.*

ensure that the study is following all aspects of the protocol.	*intervals to ensure that there is adequate documentation of critical elements such as eligibility criteria. Monitoring is required at the following timepoints (but may be done more frequently):*<br>• *study initiation*<br>• *at least every six months while participants are receiving intervention and*<br>• *annually while participants are in follow-up*<br><br>- - - - - - - - - - - -<br>All data will be reviewed by the DMC at study initiation, every six months while participants receive treatment, after the first 16 patients have completed the protocol, and annually while participants are in follow-up. A copy of the DMC charter is attached to this document.	*enrolled or the study is in long term follow up. Additional risk-based interim monitoring may be required at least once every 12-24 weeks based on the site activity, to include the possibility of remote monitoring. A longer frequency could be acceptable with justification about risk to participants.*<br><br>- - - - - - - - - -<br>All data will be reviewed by the DMC at study initiation, every six months while participants receive treatment, after the first 16 patients have completed the protocol, and annually while participants are in follow-up. Remote monitoring will be acceptable. We feel DMC review every 26 weeks is justifiable since: 1) the Study Drug is FDA-approved for >90% of children and adults with cystic fibrosis bronchiectasis, 2)	Self-assessment is acceptable.*<br>*Self-assessment*: a process for self-assessment of protocol compliance and data integrity which can be part of an overall DSMP. SeeEmory's self-assessment tool on this page.<br>- - - - - - - - - - - -<br>Ashleigh Streby and Candela Manfredi (and a 'to-be-named' Study Coordinator) will be responsible for annual self-assessment.

		the drug has been well tolerated in the CF patient population, 3) the present study cohort involves 30 subjects with evidence of partial CFTR deficiency (i.e., similar to patients already 'on label' from a safety standpoint), and 4) known toxicities of the drug primarily include increased liver function tests, rash, or other findings that will be monitored closely in the study and typically resolve if the drug is stopped.	
Real-time review of participant data during initial data collection.	Coordinators and caregivers will be trained in data acquisition, entry, and real-time review.	*Expectation is that this happens every time you obtain information.*<br>- - - - - - - - - -<br><br>Review of this type will occur at every data collection.	*Everyone on the study team responsible for primary data collection.*<br><br>William R. Hunt<br>Rebecca Kapolka<br>Rachel Linnemann<br>Arlene Stecenko<br>Ashleigh Streby<br>Colin Swenson<br>Candela Manfredi<br>'To-be-named' Study Coordinator
100% review of regulatory files	A complete review of all regulatory files will be scheduled, conducted, and signed by study coordinators. The report will be approved and	*Reviewed at a minimum of first and close-out visits*<br>- - - - - - - - - -	Ashleigh Streby<br>Candela Manfredi<br>'To-be-named' Study Coordinator

	signed by project PIs (Sorscher, Hunt).	To be conducted prior to initiation of the study and at close-out.	
100% review of consent forms	A complete review of all consent forms will be scheduled, conducted, and signed by study coordinators. The report will be approved and signed by project PIs (Sorscher or Hunt).	Prior to each DMC meeting (semiannually)	Ashleigh Streby<br>Candela Manfredi<br>'To-be-named' Study Coordinator
Review of credentials, training records, the delegation of responsibility logs (if applicable)	This will be accomplished as part of annual self-monitoring by study coordinators and the report signed by project PIs (Sorscher or Hunt).	Annually	Ashleigh Streby<br>Candela Manfredi<br>'To-be-named' Study Coordinator
Comparison of case report forms (CRF) to source documentation for accuracy and completion	This will be accomplished as part of annual self-monitoring by study coordinators and thereport signed by project PIs (Sorscher or Hunt).	Annually	Ashleigh Streby<br>Candela Manfredi<br>'To-be-named' Study Coordinator
Review of documentation of all adverse events	This will be accomplished as part of semi-annual DMC reporting by study coordinators and statistician, and the report signed by project PIs (Sorscher or Hunt).	Annually	Ashleigh Streby<br>Candela Manfredi<br>'To-be-named' Study Coordinator and Statistician
Monitoring of critical data points (eligibility, study endpoints, etc.)	This will be accomplished as part of semi-annual DMC reporting by study coordinators and statistician, and report signed by project PIs (Sorscher or Hunt).	Annually	Ashleigh Streby<br>Candela Manfredi<br>'To-be-named' Study Coordinator and Statistician
Laboratory review of processing and storage of specimens	Laboratories responsible for non-CLIA sweat chloride and CFTR genotyping, iPSC generation, and Ussing chamber analysis of airway epithelial monolayers will be internally reviewed (including protocols, processes, and SOPs relevant to this project).	*Reviewed at first and close-out visits and at least biannually*<br>- - - - - - - - - -<br>To be conducted at study initiation, biannually, and close-out.	Brian Davis, PhD or designee<br>Candela Manfredi, PhD
Assessment of laboratory specimens stored locally	Frozen iPSCs are the only biospecimens maintained for	To be conducted at study initiation,	Brian Davis, PhD, or designee

	this project, and will be monitored at UT Houston.	biannually, and close-out.	
Test article accountability review	This will be accomplished according to procedures of the Emory Research Pharmacy Investigational Drug Service (IDS)	*Reviewed at first and close-out visits and at least biannually*<br>To be conducted at study initiation, biannually, and close-out.	Per Emory IDS
Accountability logs, dispensing records, and other participant records	This will be accomplished as part of semi-annual DMC reporting by study coordinators and the report signed by project PIs (Sorscher or Hunt)	*At least biannually*<br>- - - - - - - - - -<br>Prior to each DMC meeting (biannually)	Ashleigh Streby and Candela Manfredi (and a 'to-be-named' Study Coordinator)
**For FDA regulated studies, the following requirements apply:**	**How this Requirement is Met**	**Timing, frequency, and intensity of monitoring**	**Responsible Party(ies)**
Monitoring methods (may include centralized, on-site, and self-assessment)	N/A	N/A	N/A
*For international studies, you are required to engage a CRO that is working in the site country and/or to consult with Emory's legal counsel regarding compliance with the country's clinical research regulations.			

22. Provisions to Protect the Privacy Interest of Participants

- Patient skin biopsies and iPSCs derived from skin samples will be anonymized (identifiable only by a patient-specific code). The frozen iPSCs will be maintained in liquid nitrogen at the University of Texas, Houston in a secure, key-accessible research room by Dr. B. Davis for two years after completion of the study, and then destroyed. Investigators conducting laboratory tests of iPSCs and primary airway epithelium will have access to these specimens but will not have access to patient names or identifiers.
- Data for each subject (Section 6) will be maintained in patient-specific study folders in anonymized fashion (as above) and stored in a locked and secure cabinet in a limited access study room. Patient data maintained as computer files will be password protected and maintained on a limited access, user specific network drive. All patient data specific to the study will be maintained for two years after study completion and then destroyed.
- Links between patient identification numbers and study subject data will be kept in secure form by project leadership (Sorscher and Hunt) and only made available to other study personnel as needed.

- Participant confidentiality and privacy will be strictly held in trust by the participating investigators, their staff, and others responsible for the intended interventions. This confidentiality is extended to cover testing of biological samples and other tests in addition to the clinical information relating to participants. Therefore, the study protocol, documentation, data, and all other information generated will be held in strict confidence. No confidential information concerning the study, or the data will be released to any unauthorized third party without prior written approval. Study personnel will have access to sources of information regarding participants as delineated by the informed consent document signed by all study subjects.
- All research activities will be conducted in as private a setting as possible.
- The clinical investigators, other authorized representatives, representatives of the Institutional Review Board, or regulatory agencies may inspect all documents and records required to be maintained by the investigator, including but not limited to, medical records (office, clinic, or hospital) and pharmacy records for the participants in this study. The clinical study investigators and site will permit access to such records.
- The study participant's contact information will be securely stored for internal use during the study (see above). At the end of the trial, all records will continue to be kept in a secure location for as long a period as dictated by the reviewing IRB, Institutional policies, or other requirements (currently planned for two years post-study completion).
- Study participant research data, which is for purposes of statistical analysis and scientific reporting, will be transmitted and stored securely. Individual participants and their research data will be identified by a unique study identification number. The study data entry and study management systems used by clinical research staff will be secured and password protected. At the end of the study, all study databases will be de-identified and archived by the PIs. With regard to other patient related aspects of confidentiality, all interviews and procedures will be held in a private room.
- The study team will strive to minimize any perceived intrusiveness related to questions, procedures, or testing. Our goal will be to help patients feel "at ease." Questions raised by study subjects will be addressed in a patient, non-pressured fashion, and in a manner that assures clarity and welcomes additional questioning. Extra time will be allowed to ensure patients feel at ease and comfortable with all aspects of the study.

23. Economic Burden to Participants

No costs to participants are anticipated as part of this study.

24. Informed Consent

Informed consent will be obtained by well-trained study coordinators or investigators using an Institutional Review Board approved document. A consent to allow CFTR genotyping and sweat chloride will be offered as part of the 'lead-in' study to identify potential study subjects with CFTR mutations or mildly elevated sweat chloride levels. A second consent form will be available for individuals found by the 'lead-in' study to have a single disease-causing CFTR mutation and/or sweat chloride 30-60 mEq/L, but without clinical criteria necessary for a

diagnosis of cystic fibrosis. Individuals will be asked to sign the informed consent documents only after the study has been fully described to a potential participant and all questions have been answered. Individuals will be offered at least 24 hours to review the consent before the research is to begin, although consent may be obtained on the day of screening if preferred by study subjects (e.g., those traveling from significant distance).

Consent may be obtained by any of the Emory study coordinators or caregivers listed for this protocol. The consent process will involve: 1) reading the protocol aloud together with individuals interested in participating, 2) taking particular care to answer all questions a participant might raise and addressing any questions thoroughly and patiently using language that will be easily understood, 3) allowing patients time to review informed consent materials to their satisfaction and to freely ask additional questions, and 4) allowing potential candidates as much time as needed to discuss the protocol and consent with family members or others. No pressure or sense of urgency will be employed as part of the consent process. Every effort will be made to ensure potential subjects are comfortable with their decision – and understand that they are free to decline participation without any change in their standard medical care and/or other management.

For non-English-speaking patients interested in possible study participation, interpreters fluent in the language most familiar to these individuals will work with study personnel to assure that measures under this IRB application are maintained.

25. Setting

Potential participants in this study will be recruited from the Emory University NCFBE clinic. Studies and tests (pulmonary function analysis, quality-of-life surveys, cutaneous punch biopsies, blood samples) will be performed in the Emory clinic facility. Clinical laboratory measurements will be collected during outpatient visits and analyzed in a CLIA approved laboratory at Emory. Research-related procedures such as CFTR genotyping ('lead-in' study), sweat chloride analysis ('lead-in' study), iPSC derivation, and CFTR biochemical/functional analysis may occur in the laboratories of Dr. E. Sorscher (HSRB 2nd floor, Emory University) and/or Dr. B. Davis (University of Texas, Houston). Work with UT Houston will be performed under a standard interinstitutional subcontract and statement of work approved by both Emory and UT Houston, as well as approval of the UT Houston IRB. Sweat chloride testing and genotype will be confirmed by a CLIA approved laboratory for all subjects entering the 'main' study.

26. Resources Available

The Emory NCFBE clinic follows approximately 300 individuals with NCFBE. An estimated 10-15% of these patients are expected to encode one CFTR mutation, and additional subjects to demonstrate mildly elevated sweat chloride values. The current trial intends to enroll 30 subjects with NCFBE and with a single CFTR mutation and/or elevated sweat chloride values. We expect

to recruit sufficient numbers of patients for the current trial from the Emory patient base. We also have excellent working relationships with other Atlanta and regional NCFBE pulmonary clinics, and can seek referrals from outside institutions, if needed. In either case, we plan to complete enrollment of all study subjects within a two-year project period.

The Emory NCFBE clinic comprises a state-of-the-art tertiary care healthcare center well suited to conduct all aspects of the current trial. Study Coordinators and caregivers conducting the trial will be fully accredited for performing translational work of the type described here. All study personnel will be required to read, review, and fully understand the protocol and procedures associated with the trial, and will be familiarized with their roles and responsibility as part of this project.

27. References

Barillà, C., S. Suzuki, A. Rab, B. Wang, J. Hong, W. Driggers, A. Streby, R. Feldman, R. Linnemann, G. Solomon, A. Stecenko, E. Sorscher and B. Davis (2021). "667: Development of an iPSC-based airway epithelial platform for evaluating patient-specific responses to modulators." Journal of Cystic Fibrosis **20**: S316.

Barry, P. J., M. A. Mall, D. Polineni and V. X. S. Group (2021). "Triple Therapy for Cystic Fibrosis Phe508del-Gating and -Residual Function Genotypes. Reply." N Engl J Med **385**(23): 2208.

Boyle, M. P., S. C. Bell, M. W. Konstan, S. A. McColley, S. M. Rowe, E. Rietschel, X. Huang, D. Waltz, N. R. Patel, D. Rodman and V. X. s. group (2014). "A CFTR corrector (lumacaftor) and a CFTR potentiator (ivacaftor) for treatment of patients with cystic fibrosis who have a phe508del CFTR mutation: a phase 2 randomised controlled trial." Lancet Respir Med **2**(7): 527-538.

Brewington, J., J. Hong, C. Manfredi, A. Rab, D. Joshi, R. Linnemann, A. Streby, A. Stecenko, A. Ostmann, R. O'Shaughnessy, H. Morgan, J. Meeker, S. Suzuki, C. Barillà, B. Wang, Y. Cheng, H. Bihler, K. Coote, B. Davis, M. Mense, G. Solomon and E. Sorscher (2021). "638: Demonstration of pharmacologic N1303 K CFTR rescue in heterologous and human tissue-based model systems." Journal of Cystic Fibrosis **20**: S303.

Cabrini, G., A. Rimessi, M. Borgatti, I. Lampronti, A. Finotti, P. Pinton and R. Gambari (2020). "Role of Cystic Fibrosis Bronchial Epithelium in Neutrophil Chemotaxis." Front Immunol **11**: 1438.

Casals, T., J. De-Gracia, M. Gallego, J. Dorca, B. Rodriguez-Sanchon, M. D. Ramos, J. Gimenez, A. Cistero-Bahima, C. Olveira and X. Estivill (2004). "Bronchiectasis in adult patients: an expression of heterozygosity for CFTR gene mutations?" Clin Genet **65**(6): 490-495.

Chalmers, J. D., A. B. Chang, S. H. Chotirmall, R. Dhar and P. J. McShane (2018). "Bronchiectasis." Nat Rev Dis Primers **4**(1): 45.

Crane, A. M., P. Kramer, J. H. Bui, W. J. Chung, X. S. Li, M. L. Gonzalez-Garay, F. Hawkins, W. Liao, D. Mora, S. Choi, J. Wang, H. C. Sun, D. E. Paschon, D. Y. Guschin, P. D. Gregory, D. N. Kotton, M. C. Holmes, E. J. Sorscher and B. R. Davis (2015). "Targeted correction and restored function of the CFTR gene in cystic fibrosis induced pluripotent stem cells." Stem Cell Reports **4**(4): 569-577.

Ebert, A. D., P. Liang and J. C. Wu (2012). "Induced pluripotent stem cells as a disease modeling and drug screening platform." J Cardiovasc Pharmacol **60**(4): 408-416.

Flume, P. A., J. D. Chalmers and K. N. Olivier (2018). "Advances in bronchiectasis: endotyping, genetics, microbiome, and disease heterogeneity." Lancet **392**(10150): 880-890.

Hawkins, F., P. Kramer, A. Jacob, I. Driver, D. C. Thomas, K. B. McCauley, N. Skvir, A. M. Crane, A. A. Kurmann, A. N. Hollenberg, S. Nguyen, B. G. Wong, A. S. Khalil, S. X. Huang, S. Guttentag, J. R. Rock, J. M. Shannon, B. R. Davis and D. N. Kotton (2017). "Prospective isolation of NKX2-1-expressing human lung progenitors derived from pluripotent stem cells." J Clin Invest **127**(6): 2277-2294.

Jacob, A., M. Morley, F. Hawkins, K. B. McCauley, J. C. Jean, H. Heins, C. L. Na, T. E. Weaver, M. Vedaie, K. Hurley, A. Hinds, S. J. Russo, S. Kook, W. Zacharias, M. Ochs, K. Traber, L. J. Quinton, A. Crane, B. R. Davis, F. V. White, J. Wambach, J. A. Whitsett, F. S. Cole, E. E. Morrisey, S. H. Guttentag, M. F. Beers and D. N. Kotton (2017). "Differentiation of Human Pluripotent Stem Cells into Functional Lung Alveolar Epithelial Cells." Cell Stem Cell **21**(4): 472-488 e410.

Linnemann, R., G. Solomon, A. Streby, A. Rab, W. Driggers, K. Slaten, H. Hathorne, J. Hong, S. Suzuki, B. Wang, C. Barillà, A. Stecenko, B. Davis and E. Sorscher (2021). "572: Clinical and iPSC-derived airway epithelial responses to elexacaftor/tezacaftor/ivacaftor in CF patients without an approved modulator." Journal of Cystic Fibrosis **20**: S271.

Lopes-Pacheco, M. (2019). "CFTR Modulators: The Changing Face of Cystic Fibrosis in the Era of Precision Medicine." Front Pharmacol **10**: 1662.

Manfredi, C., J. M. Tindall, J. S. Hong and E. J. Sorscher (2019). "Making precision medicine personal for cystic fibrosis." Science **365**(6450): 220-221.

Maselli, D. J., B. Amalakuhan, H. Keyt and A. A. Diaz (2017). "Suspecting non-cystic fibrosis bronchiectasis: What the busy primary care clinician needs to know." Int J Clin Pract **71**(2).

Middleton, P. G., M. A. Mall, P. Drevinek, L. C. Lands, E. F. McKone, D. Polineni, B. W. Ramsey, J. L. Taylor-Cousar, E. Tullis, F. Vermeulen, G. Marigowda, C. M. McKee, S. M. Moskowitz, N. Nair, J. Savage, C. Simard, S. Tian, D. Waltz, F. Xuan, S. M. Rowe, R. Jain and V. X. S. Group (2019). "Elexacaftor-Tezacaftor-Ivacaftor for Cystic Fibrosis with a Single Phe508del Allele." N Engl J Med **381**(19): 1809-1819.

Miller, A. C., A. P. Comellas, D. B. Hornick, D. A. Stoltz, J. E. Cavanaugh, A. K. Gerke, M. J. Welsh, J. Zabner and P. M. Polgreen (2020). "Cystic fibrosis carriers are at increased risk for a wide range of cystic fibrosis-related conditions." Proc Natl Acad Sci U S A **117**(3): 1621-1627.

Mou, H., K. Brazauskas and J. Rajagopal (2015). "Personalized medicine for cystic fibrosis: establishing human model systems." Pediatr Pulmonol **50 Suppl 40**: S14-23.

Pignatti, P. F., C. Bombieri, C. Marigo, M. Benetazzo and M. Luisetti (1995). "Increased incidence of cystic fibrosis gene mutations in adults with disseminated bronchiectasis." Hum Mol Genet **4**(4): 635-639.

Polgreen, P. M. and A. P. Comellas (2022). "Clinical Phenotypes of Cystic Fibrosis Carriers." Annu Rev Med **73**: 563-574.

Suzuki, S., A. M. Crane, V. Anirudhan, C. Barilla, N. Matthias, S. H. Randell, A. Rab, E. J. Sorscher, J. L. Kerschner, S. Yin, A. Harris, M. Mendel, K. Kim, L. Zhang, A. Conway and B. R. Davis (2020). "Highly efficient gene editing of cystic fibrosis patient-derived airway basal cells results in functional CFTR correction." Mol Ther **28**(7): 1684-1695.

Vermeulen, F., C. Le Camus, J. C. Davies, D. Bilton, D. Milenkovic and K. De Boeck (2017). "Variability of sweat chloride concentration in subjects with cystic fibrosis and G551D mutations." J Cyst Fibros **16**(1): 36-40.

Weycker, D., G. L. Hansen and F. D. Seifer (2017). "Prevalence and incidence of noncystic fibrosis bronchiectasis among US adults in 2013." Chron Respir Dis **14**(4): 377-384.

Figure S1 (Supplemental Materials)

Trikafta can improve lung function in a patient with low positive sweat chloride and no known CFTR mutations

- Case study – unpublished study

  56-year-old male with diagnosis of CF based on:
  - Elevated SwCl in the 60s (mEq/L, >60 = indicative of CF)
  - Severe bronchiectasis (FEV_1 ~40 percent of predicted) with pronounced daily respiratory symptoms
  - Chronic sinusitis
  - Mild pancreatic exocrine insufficiency
- Very significant improvement in FEV_1 after 28 days of Trikafta™ (+20%) with evidence of CFTR activation, improvement in quality of life/respiratory symptom score, and weight gain

Efficacy Data

Age	Mutation	ΔFEV_1 from Baseline to Day 28 (% predicted)	ΔSwCl from Baseline to Day 28 (mEq/L)	ΔCFQ-R respiratory domain from Baseline to Day 28
56	None detected/None detected	+20	-12	+5

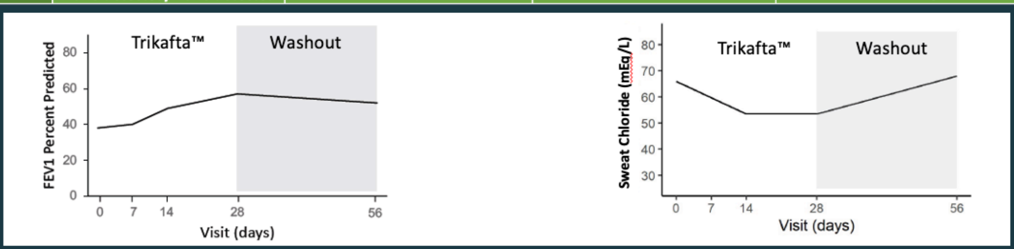

Changes in Clinical Outcomes with 28 days of Trikafta™ treatment (shading signifies "wash-out" period). CFQ-R minimum clinically important difference: 4 points

28. Protocol Checklist

Please note that protocol sections with an asterisk (*) should always be included in the protocol; if the section does not have an asterisk, and you have not included the section in the protocol, the IRB will consider it your attestation that the section does not apply to your study.

**Protocol Section**	**Added to the protocol?**
**External Collaborators**- if applicable, add each external collaborator information and indicate whether that institution's IRB will review (or has already reviewed) that individual's engagement in human participants research activities)	☒ **Yes**
**Funding Source***: Include the information for the funding entity for this study. Please explain if this study is covered by a sub-award or other pertinent information. Say "department" if you do not have any other funding.	☒ **Yes**
**Objectives*:** Describe the purpose, specific aims, or objectives and state the hypotheses to be tested	☒ **Yes**
**Background*:** Describe the relevant prior experience and gaps in current knowledge. Describe any relevant preliminary data. Provide the scientific or scholarly background for, the rationale for, and significance of the research based on the existing literature and how will it add to existing knowledge	☒ **Yes**
**Study Endpoints*:** Describe the primary and secondary study endpoints. Describe any primary or secondary safety endpoints.	☒ **Yes**
**Study Intervention/Investigational Agent*:** Describe the study intervention and/or investigational agent (e.g., drug, device) that is being evaluated.	☒ **Yes**
**Drug/Device Handling:** If the research involves drugs or devices, describe your plans to store, handle, and administer those drugs or devices so that they will be used only on participants and be used only by authorized investigators.<br>If using a drug, explain if the control of the drug is managed by IDS (or VA/Grady/CHOA research pharmacies). If not, provide IDS exemption document.<br>If a device, explain how the device is being stored and managed.	☒ **Yes**
If the drug is under an FDA REMS, plan to complete the REMS checklist found here, on the IRB website.	☐ **Yes**
If the drug is considered a controlled substance, make sure you have filled out this form.	☐ **Yes**

If applicable, identify the holder of the IND/IDE/Abbreviated IDE. An Emory investigator who holds an IND or IDE is considered to be a Sponsor-Investigator (S-I). If the study is under an S-I, review this section of our website for additional requirements.	☐ **Yes**
**Procedures involved***: Describe and explain the study design and include a study schema. Describe all research procedures being performed and when they are performed, including procedures being performed to monitor participants for safety or minimize risks	☒ **Yes**
**Procedures-Minimizing risk*:** describe the procedures performed to lessen the probability or magnitude of risks.	☒ **Yes**
**Procedures- Drug/Device Use:** describe all drugs and devices used in the research and the purpose of their use and their regulatory approval status	☒ **Yes**
**Procedures-Source Records*:** describe source records that will be used to collect data about participants. Attach all surveys, scripts, and data collection forms to the submission.	☒ **Yes**
**Procedures-Data collection*:** describe what data will be collected during the study and how that data will be obtained	☒ **Yes**
**Procedures- Long Term Follow Up*:** once all research-related procedures are complete, what data will be collected during this period. If no data is collected after procedures are completed, please state in the submission.	☒ **Yes**
**Data and Specimen Banking:** describe where the specimens will be stored, how long they will be stored, how the specimens will be accessed, and who will have access to the specimens. Depending on the volume and nature of the collection, this may require a separate repository-specific IRB submission. The VA Data Repository SOP is required if the study is creating a data repository at the Atlanta VA.<br>List the data to be stored or associated with each specimen.<br>Describe the procedures to release data or specimens, including the process to request a release, approvals required for release, who can obtain data or specimens, and the data to be provided with specimens.	☒ **Yes**
**Sharing of Results with Participants*:** Describe whether results (study results or individual subject results, such as results of investigational diagnostic tests, genetic tests, or incidental findings) will be shared with participants or others (e.g., the participant's primary care physicians) and if so, describe how the results will be shared If applicable (e.g. for studies involving scans and/or panels of exploratory testing on specimens)	☒ **Yes**

Plan for managing the types of findings that might arise. This should include any secondary findings that are being sought actively, findings that might be anticipatable, and findings that might be un-anticipatable.<br>Plan for recognizing, analyzing, and handling incidental findings and how incidental findings will be communicated to participants during the consent process. If the plan is not to disclose any findings, then this should be included. This plan might include the option for participants to opt-out of receiving incidental findings.<br>Description of the research team's responsibilities following disclosure of a finding. This should detail educational information about the nature of the finding, how to seek care from a clinician or specialist, obtaining health insurance to secure treatment, and/or referral to a clinical specialist, if one is required.<br>Reminder to include language in the consent form to let the participants know your plans for this – see Modular Language for Informed Consent Forms on IRB website)	
**Study timelines*:** describe the duration of an individual participant's participation in the study; anticipated time to enroll all study participants and the estimated date for the investigators to complete this study (complete primary analyses)	☒ **Yes**
**Inclusion and Exclusion Criteria*:** describe how individuals will be screened for eligibility and the criteria that define who will be included or excluded in your final study sample	☒ **Yes**
**Population*:** describe the study popualation and indicate specifically whether you will include or exclude each of the following special populations:<br>• Adults unable to consent<br>• Individuals who are not yet adults (infants, children, teenagers)<br>• Pregnant women<br>• Prisoners<br><br>Note: you cannot exclude people with limited English proficiency unless you can demonstrate the scientific need for such exclusion.<br>Community Participation: For studies aimed at addressing issues that affect a certain community or group: How, if at all, will this study involve people from the target community in the design of the study? Conduct of the study? How will the results of the research be shared with the participants and/or the target community/ies?<br><br>**If studying Race or Ethnicity, have you defined these terms, and explained their proposed mechanism of action if these characteristics will be used in an explanatory model?**	☒ **Yes**
**Research with pregnant women, fetuses, or neonates:** review this checklist to verify you have provided enough information to ensure the safety and well-being of this population.	☐ **Yes**

**Research with neonates of uncertain viability:** review this checklist to verify you have provided enough information to ensure the safety and well-being of this population.	☐ **Yes**
**Research involving prisoners:** review this checklist to verify you have provided enough information to ensure the safety and well-being of this population.	☐ **Yes**
**Research involving children:** review this checklist to verify you have provided enough information to ensure the safety and well-being of this population.	☐ **Yes**
**Research involving cognitively impaired adults:** review this checklist to verify you have provided enough information to ensure the safety and well-being of this population.	☐ **Yes**
**Research involving economically or educationally disadvantaged persons:** describe the additional safeguards that have been included in the study to protect the rights and welfare of these subjects	☐ **Yes**
**Local Number of Participants*:** Indicate the total number of participants to be accrued locally. If applicable, distinguish between the number of participants who are expected to be enrolled and screened, and the number of participants needed to complete the research procedures (i.e., numbers of participants excluding screen failures.)<br>Provide your projected enrolling goals, including the percentage of participants according to sex and race.	☒ **Yes**
**Recruitment Methods*:** Describe when, where, and how potential participants will be recruited. Describe the source of participants. Describe the methods that will be used to identify potential participants. Describe materials that will be used to recruit participants. Attach copies of these documents with the application.<br>If including advertisements, attach the final copy of them. When advertisements are taped for broadcast*, attach the final* audio/videotape. You may submit the wording of the advertisement before taping to preclude re-taping because of inappropriate wording, provided the IRB reviews the final audio/videotape. Describe the amount and timing of any payments to participants. Reimbursement for expenses/travel?<br>If using contests or raffles as incentive, you must offer entry to all potential participants, not just those who enroll in the study/complete study-related procedures, per Georgia State Law.<br>All research recruitment through social media needs to follow this guidance, which does not allow the use of personal social media accounts for some recruitment activities.	☒ **Yes**
**Withdrawal of Participants*:** Describe anticipated circumstances under which participants will be withdrawn from the research without their consent. Describe any procedures for	☒ **Yes**

orderly termination. Describe procedures that will be followed when participants withdraw from the research, including partial withdrawal from procedures with continued data collection.	
**Risk to Participants*:** List the reasonably foreseeable risks, discomforts, hazards, or inconveniences to the participants related to the participant's participation in the research. Include as may be useful for the IRB's consideration, a description of the probability, magnitude, duration, and reversibility of the risks. Consider physical, psychological, social, legal, and economic risks.<br>If applicable, indicate which procedures may have risks to the participants that are currently unforeseeable.<br>If applicable, indicate which procedures may have risks to an embryo or fetus should the subject be or become pregnant.<br>If applicable, describe risks to others who are not participants.	☒ **Yes**
**Potential Benefits to Participants*:** Describe the potential benefits that individual participants may experience from taking part in the research. Include as may be useful for the IRB's consideration, the probability, magnitude, and duration of the potential benefits. Indicate if there is no direct benefit. Do not include benefits to society or others.	☒ **Yes**
**Compensation to Participants*:** Describe if/how subjects will be compensated for participation in this study. Indicate what method compensation will be delivered (e.g. cash, gift card, school credit). Describe the amount and timing of any payments to participants. How much? What kind? Is tax information required? (if so, must be reflected in the informed consent form). Will payments be pro-rated if a participant withdraws early?	☒ **Yes**
**Data Management and Confidentiality*:** Describe the data analysis plan, including any statistical procedures or power analysis. Describe the steps that will be taken to secure the data (e.g., training, authorization of access, password protection, encryption, physical controls, certificates of confidentiality, and separation of identifiers and data) during storage, use, and transmission. Describe any procedures that will be used for the quality control of collected data.	☒ **Yes**
**Describe how data or specimens will be handled study-wide*:** What information will be included in that data or associated with the specimens?<br>• Where and how data or specimens will be stored?<br>• How long the data or specimens will be stored?<br>• Who will have access to the data or specimens?<br>• Who is responsible for receipt or transmission of the data or specimens?<br>• How data or specimens will be transported?	☒ **Yes**

**Data Monitoring and Participants Safety (if this study is more than minimal risk, this section is required):**	☒ **Yes**
Ensure that you review our Data and Safety Monitoring plan guidance for specific details about this section, and examples of what the IRB will be requiring according to the level of risk.<br><br>If a DSMB is needed, please describe the composition of the board (if not already detailed in the protocol). Review this guidance for more information. If the sponsor protocol does not contain all required information, please in this section.<br><br>Describe the plan to periodically monitor the data at the site level according to risk level. Include the appropriate completed monitoring table, if applicable.<br><br>Description of the plan for notifying the IRB of reportable events, whether the sponsor requires reporting above and beyond the Emory IRB reporting requirements, and if so, a description of the requirements and plan for meeting them.<br><br>Please address the specific details below. If deemed not applicable, please provide rationale:<br><br>Subject safety:<br>• Specific subject safety parameters<br>• Frequency of subject safety observations<br>• Individual responsible for safety monitoring<br>• Subject stopping rules – under what conditions will a subject be removed from study participation and who will make the decision?<br>• Study stopping rules - under what conditions will the study be modified or stopped and who will make the decision?<br>• Reporting mechanisms (i.e. Deviations, adverse events, UPs)<br><br>Data Integrity:<br>• Specific data elements to be reviewed<br>• Frequency of monitoring data, points in time, or after a specific number of participants<br>• Individual responsible for data monitoring<br><br>Additional considerations for FDA regulated trials<br><br>Depending on the procedures affecting risks to participants, the site monitoring plan should specify:	

• Categorization of activities done centrally and those on-site if applicable<br>• Monitoring methods (may include centralized/remote, on-site, and self-monitoring)<br>• Reference to any tools used (i.e. checklists)<br>• Identification of events that may trigger changes<br>• Identification of deviations or failures that would be critical to study integrity	
**Provisions to Protect the Privacy Interests of Participants*:**<br>• Describe the steps that will be taken to protect participants' privacy interests. "Privacy interest" refers to a person's desire to place limits on whom they interact with or whom they provide personal information.<br>• Describe what steps you will take to make the participants feel at ease with the research situation in terms of the questions being asked and the procedures being performed. "At ease" does not refer to physical discomfort, but the sense of intrusiveness a participant might experience in response to questions, examinations, and procedures.<br>• Indicate how the research team is permitted to access any sources of information about the participants.	☒ **Yes**
**Economic Burden to Participants*:** Describe any costs that participants may be responsible for because of participation in the research.	☒ **Yes**
**Consent Process*:** Describe where the consent process will take place, any waiting period available between informing the prospective subject and obtaining the consent; and the process to ensure ongoing consent.<br>Describe the role of the individuals listed in the application as being involved in the consent process; the time that will be devoted to the consent discussion; steps that will be taken to minimize the possibility of coercion or undue influence; and steps that will be taken to ensure the participants' understanding.<br>**Note**: If you are planning to obtain consent via electronic signature, please review this document. Additional guidance on consent documentation and process can be found on our website, under the consent toolkit.	☒ **Yes**
**Consent Process-Non-English-Speaking Participants*:**<br>Indicate what language(s) other than English are understood by prospective participants or representatives.<br>If participants who do not speak English will be enrolled, describe the process to ensure that the oral and written information provided to those participants will be in that language. Indicate the language that will be used by those obtaining consent.<br>If you checked N/A, please provide reasoning of why subjects with limited English proficiency are excluded.	☒ **Yes**

**Note**: if you stated that subjects with LEP will be enrolled, you are approved for the use of the Emory IRB short forms. Please read the guidance about the use of short forms here.	
**Consent Process-Children:** After determining if the subject is a child per GA law (or if enrolled outside GA, per state/country law), please describe whether parental permission will be obtained from:	☐ **Yes**
• Both parents unless one parent is deceased, unknown, incompetent, or not reasonably available, or when only one parent has legal responsibility for the care and custody of the child.<br>• One parent even if the other parent is alive, known, competent, reasonably available, and shares legal responsibility for the care and custody of the child.	
Describe whether permission will be obtained from individuals other than parents, and if so, who will be allowed to provide permission. Describe the process used to determine these individuals' authority to consent to each child's general medical care.	
When assent of children is obtained describe whether and how it will be documented per Emory Policies and Procedures	
**Consent Process-Cognitively Impaired Adults:** describe the process to determine whether an individual is capable of consent. The IRB allows the person obtaining assent to document assent on the consent document and does not routinely require assent documents and does not routinely require children to sign assent documents.	☐ **Yes**
**Consent Process-Adults Unable to Consent:** List the individuals from whom permission will be obtained in the order of priority. (E.g., durable power of attorney for health care, a court-appointed guardian for health care decisions, spouse, and adult child.)<br>For research conducted in the state, review "46 LEGALLY AUTHORIZED REPRESENTATIVES AND SURROGATE CONSENT" to be aware of which individuals in the state meet the definition of "legally authorized representative."<br>For research conducted outside of the state, provide information that describes which individuals are authorized under applicable law to consent on behalf of a prospective subject to their participation in the procedure(s) involved in this research.<br>Describe the process for the assent of the participants. Indicate whether:	☐ **Yes**
• Assent will be required of all, some, or none of the participants. If some, indicated, which participants will be required to assent and which will not.<br>• If assent will not be obtained from some or all participants, an explanation of why not.	

Describe whether the assent of the participants will be documented and the process to document assent. The IRB allows the person obtaining assent to document assent on the consent document and does not routinely require assent documents and does not routinely require participants to sign assent documents	
**Waiver or Alteration of Consent Process (consent will not be obtained, required information will not be disclosed, or the research involves deception)**<br>Review the Emory IRB waiver document to ensure you have provided sufficient information for the IRB to make these determinations.<br>If the research involves a waiver of the consent process for planned emergency research, please review the "CHECKLIST: Waiver of Consent for Emergency Research (HRP-419)" to ensure you have provided sufficient information for the IRB to make these determinations.	☐ **Yes**
**Setting*:** Describe the sites or locations where your research team will conduct the research including where the subject will be identified and recruited, where the research procedures will be performed, and if you will involve a community advisory board. For research conducted outside the organization and its affiliates describe the site-specific regulations or customs affecting the research outside the organization and the local scientific and ethical review structure outside the organization.	☒ **Yes**
**Resources Available*:** Describe the resources available to conduct the research such us the feasibility of recruiting the required number of suitable participants within the agreed recruitment period; describe the time that you will devote to conducting and completing the research; describe the availability of medical or psychological resources that participants might need as a result of an anticipated consequences of the human research; describe your process to ensure that all persons assisting with the research are adequately informed about the protocol, the research procedures, and their duties and functions.	☒ **Yes**
**Multi-Site Research when Emory is the Lead Site:**<br>Study -Wide Number of Participants: indicate the total number of participants to be accrued across all sites.<br>Study-Wide Recruitment Methods: If this is a multicenter study and participants will be recruited by methods not under the control of the local site (e.g., call centers, national advertisements) describe those methods.<br>Describe when, where, and how potential participants will be recruited.<br>Describe the methods that will be used to identify potential participants.<br>Describe materials that will be used to recruit participants.<br>Describe the processes to ensure communication among sites. See "WORKSHEET: Communication and Responsibilities (HRP-830)." All sites have the most current version of the protocol, consent document, and HIPAA authorization.	☐ **Yes**

All required approvals (initial, continuing review and modifications) have been obtained at each site (including approval by the site's IRB of record).<br>All modifications have been communicated to sites and approved (including approval by the site's IRB of record) before the modification is implemented.<br>All engaged participating sites will safeguard data, including secure transmission of data, as required by local information security policies.<br>All local site investigators conduct the study in accordance with applicable federal regulations and local laws.<br>All non-compliance with the study protocol or applicable requirements will reported in accordance with local policy<br>Describe the method for communicating to engaged participating sites (see "WORKSHEET: Communication and Responsibilities (HRP-830)"):	
• Problems (inclusive of reportable events).<br>• Interim results.<br>• The closure of a study	
If this is a multicenter study where you are a participating site/investigator, describe the local procedures for maintenance of confidentiality. (See "WORKSHEET: Communication and Responsibilities (HRP-830).")	
• Where and how data or specimens will be stored locally?<br>• How long the data or specimens will be stored locally?<br>• Who will have access to the data or specimens locally?<br>• Who is responsible for receipt or transmission of the data or specimens locally?<br>• How data and specimens will be transported locally?	
