# Supplementary material for: Evaluating elexacaftor/tezacaftor/ivacaftor (ETI; Trikafta™) for treatment of patients with non-cystic fibrosis bronchiectasis (NCFBE): A clinical study protocol
Source: PLoS One. 2025 Feb 14;20(2):e0316721. doi: 10.1371/journal.pone.0316721 (PMC11828409; doi:10.1371/journal.pone.0316721)
Supplement: S2 File — (PDF) [file pone.0316721.s003.pdf]

You Are Being Asked to Be in a Research Study

Concise presentation of key concepts

You are being asked to be in a research study. A research study is designed to answer a scientific question. If you agree to be in the study, you will be one of approximately 200 people who are being studied at Emory.

Why is this study being done?
This study is being done to answer the question: How many patients at Emory clinics exhibit clinical features of non-cystic fibrosis bronchiectasis (NCFBE) and may benefit from Trikafta™? You are being asked to be in this research study because you are a patient at Emory and you have been diagnosed with NCFBE.

Do you have to be in the study?
It is your choice to join this research study. You do not have to be in it. Your choice will not affect your access to medical care for your condition. Before you choose, take time to learn about the study.

What do you have to do if you choose to join this study?
If you qualify and choose to join the study, you will participate for 2-3 hours (1 study visit). The researchers will ask you to do the following: 1) undergo a blood test to determine whether you have mutations in your CFTR gene, and 2) have sweat collected from your forearm to measure the level of chloride. ALL of these procedures will be paid for by the study.

How is this study going to help you?
If you are in the study, you will be helping the researchers answer the study question.

What are the risks or discomforts you should know about before deciding?
The study will take time. All studies have some risks. Some risks are relatively small, like being bored or losing time. Some are more serious. Risks for this study include:

- blood draws have the risk of hematoma (bruising) at the needle site and minimal pain due to the venous puncture procedure
- sweat chloride testing: the Macroduct system is used in hospitals and clinics worldwide to perform sweat chloride testing. This is considered a very safe, painless procedure, but rare minor burns have been reported at the electrode site (although participants may show no discomfort during the test). To further minimize risk, all equipment will be

properly cleaned and evaluated before and after the procedure by personnel trained in performance of this assay.

- loss of privacy
- breach of confidentiality

You can find a full list of expected risks, their frequency and severity in the section titled "What are the possible risks and discomforts?"

Alternatives to Joining This Study
Since this is not a treatment study, the alternative is not to participate.

Costs
There will be no costs to you for participating in this study, other than basic expenses like transportation. You will not be charged for any of the research activities.

There is more information in the "Costs" section further below.

What Should You Do Next?
Read this form, and/or have it read to you. Make sure the study doctor or study staff explains the study to you. Ask questions such as how much time you will have to spend on the study, any words you do not understand and more details about study procedures. Take time to think about this and talk about it with your family and friends.

Emory University
Consent to be a Research Subject / HIPAA Authorization

Title: Evaluating Trikafta for the treatment of patients with non-cystic fibrosis bronchiectasis (NCFBE)
Consent for CFTR genotype and sweat chloride analysis (Lead-in study)

IRB #: 00004736

Principal Investigators: Eric Sorscher, MD, Department of Pediatrics; Randy Hunt, MD, Department of Medicine; Colin Swenson, MD, Department of Medicine

Study-Supporter: The Marcus Foundation

Introduction

You are being asked to be in a medical research study. This form tells you what you need to think about before you choose if you want to join the study. **It is your choice. If you choose to join, you can change your mind later and leave the study.** Your choice will not cause you to lose any medical benefits. If you choose not to join this study, your doctor will still treat you.

Before you decide:

- Read this form or have it read to you
- Listen to the study doctor or study staff explain the study to you
- Ask questions about anything that is not clear

You will get a copy of this form. Take your time to think about joining the study. You may wish to discuss it with family or friends. Do not sign this form if you still have questions or something does not make sense to you. By signing this form, you will not give up any legal rights.

What is the purpose of this study?

Non-cystic fibrosis bronchiectasis (NCFBE) is a chronic, progressive lung disorder characterized by shortness of breath, wheezing, persistent cough, excessive phlegm production and recurrent lung infections. NCFBE is similar to cystic fibrosis (CF) lung disease – but patients with NCFBE do not exhibit all of the symptoms of CF. Trikafta™ is an oral drug that improves lung function in CF. Trikafta™ is approved for patients with CF but has not been studied in patients with NCFBE. This project is intended to test whether certain patients with NCFBE (also simply called "bronchiectasis") may benefit from Trikafta™. We believe patients with NCFBE who have: 1) 1 mutation in a gene called the cystic fibrosis transmembrane conductance regulator (CFTR) and/or 2) slightly elevated levels of chloride in sweat will be those most likely to exhibit improved lung function on Trikafta™. The purpose of this part of our study is to identify patients at Emory clinics who exhibit clinical features and may benefit from Trikafta™.

What will you be asked to do?

If you qualify for the study, you will: 1) undergo a blood test to determine whether you have mutations in your CFTR gene, and 2) have sweat collected from your forearm to measure the level of chloride.

You will need to take part in 1 office visit (2-3 hours) to complete this study. We hope to enroll patients who are ages 18 years old and older and seen at Emory with a diagnosis of NCFBE. The following tests will be done. Both will be explained later in the consent and again in detail by the study Doctor.

- CFTR Genotyping (to determine whether you have a CFTR mutation)
- Sweat Chloride Collection

Blood Draw/CFTR mutations test:
About 1 tablespoon of blood will be taken from your arm for checking CFTR mutations.

Sweat Chloride Test:
In the sweat test, a place on your arm is stimulated with electrodes to produce sweat. The sweat is caught in a collector disc placed on the skin. We will measure chemicals, such as salt, in your sweat. To collect the sweat, 2 probes will be attached to the skin on your arm for 4-5 minutes. A gel-like medicine called pilocarpine is put on the probes and causes the sweat glands to produce more sweat. The probes stay on for about 4-5 minutes, then are removed and replaced by a disc to collect sweat for about 30 minutes. The entire procedure will then be repeated on the other arm.

> Note: The research team for this study includes non-licensed team members who may obtain your consent, or help guide you through the study. There are some kinds of questions only licensed clinicians can answer. For example, detailed questions about drug interactions. If you have questions like these, the non-licensed coordinator will ask a licensed study team member to answer your questions.

Who owns your study data and samples?
If you join this study, you will be donating your samples and data. You will not be paid if your samples or data are used to make a new product. If you leave the study, the data and samples that were already collected may be still be used for this study.

What are the possible risks and discomforts?
The most common risks and discomforts expected in this study are:

- Blood draw: This is the standard medical method used to obtain blood for tests. There is momentary pain at the time the needle is inserted into the vein, but other discomfort should be minimal. In about 10% of the cases there is a small amount of bleeding under the skin which will produce a bruise. The risk of infection is less than 1 in 1,000.

Rare but possible risks include:

- Sweat chloride testing: the Macroduct system is used in hospitals and clinics worldwide to perform sweat chloride testing. This is considered a very safe, painless procedure, but rare minor burns (like a sunburn) have been reported at the electrode site (although participants may show no discomfort during the test). The risk is less than 1 in 50,000. To further minimize risk, all equipment will be properly cleaned and evaluated before and after the procedure by personnel trained in performance of this assay.

Researchers may learn something new during the study that may affect your choice to be in the study. If this happens, they will tell you about it. Then you can choose if you want to stay in this study. You may be asked to sign a new form if you choose to stay in the study.

Researchers may also learn something new about your underling health condition. This could include determining that you have a diagnosis of cystic fibrosis, a cystic fibrosis related condition, or a risk of developing cystic fibrosis in your family. If you complete the study visit, we will share your study data with your NCFBE doctor who can help you interpret the results.

Will you benefit from the study?
This study is not designed to benefit you directly. Patients with NCFBE are more likely to show evidence of a CFTR mutation. Approximately 1 of 30 Caucasian Americans without NCFBE exhibit a CFTR mutation. If we identify patients with NCFBE and a CFTR mutation and/or mildly increased sweat chloride, we will be in a position to ask whether some of these patients would be willing to receive Trikafta™ as a potential new treatment. In this way, the study results may help others in the future.

Will you be paid for your time and effort?

Travel, meals, accommodations, etc. for study visits will be paid for by the study as needed and an incentive payment of $150 will be provided. For adult subjects who are accompanied by a caregiver, travel expenses, meals, and accommodations may also be provided for the caregiver.

You will be asked to fill out a tax form, including your Social Security or Taxpayer Identification Number, in order to be reimbursed, depending on the amount and method of payment. Some payment methods involve mail coming to your house, which may be seen by others in your household. You can decline payment if you are concerned about confidentiality, or you can talk to the study team to see if there are other payment options.

What are your other options?
If you decide not to enter this study, there is care available to you outside of this research study. This includes all aspects of ongoing and standard care with your usual NCFBE clinical team and care center. You do not have to be in this study to be treated for NCFBE.

Taking part in this study may make you unable to participate in some other research studies, if they exclude people who have mutations in CFTR or elevated sweat chloride. You should discuss this with the researchers if you have concerns. You may wish to research other study options at websites like clinicaltrials.gov or ResearchMatch.org.

How will your private information be protected?
Whenever possible, a study number, rather than your name, will be used on study records. Your name and other identifying information will not appear when we present or publish the study results.

Study records can be opened by court order. They also may be provided in response to a subpoena or a request for the production of documents.

Storing and Sharing your Information
We will store all the data and specimens that you provide using a code. We need this code so that we can keep track of your data over time. This code will not include information that can identify you (identifiers). Specifically, it will not include your name, initials, date of birth, or medical record number. We will keep a file that links this code to your identifiers in a secure location separate from the data.

We will not allow your name and any other fact that might point to you to appear when we present or publish the results of this study.

Your data from this study, including your de-identified genetic information, may be shared with the research community at large to advance science and health. Data from this study may be placed into public databases where, in addition to having no direct identifiers, researchers will need to sign data use agreements before accessing the data. We will remove or code any personal information that could identify you before your information is shared. This will ensure

that, by current scientific standards and known methods, it is extremely unlikely that anyone would be able to identify you from the information we share. Despite these measures, we cannot guarantee anonymity of your personal data.

We will use your specimens and data only for research. We will not sell them. However, the results of this research might someday lead to the development of products (such as a commercial cell line, a medical or genetic test, a drug, or other commercial product) that could be sold by a company. You will not receive money from the sale of any such product.

Returning Results to Participants/Incidental Findings
After you complete the study visit, we will share your study data with your NCFBE doctor who can help you interpret the results.

Medical Record
If you have been an Emory patient before, then you already have an Emory medical record. If you have never been an Emory patient, you do not have one. An Emory medical record will be made for you if an Emory Atlanta provider or facility gives you any services or procedures for this study.

Copies of the consent form/HIPAA authorization that you sign will be put in any Emory medical record you have now or any time during the study.

The results of some study tests and procedures will be used only for research purposes and will *not* be placed in your medical record. For this study, those items include: results of genotyping and a sweat test.

Tests and procedures done at non-Emory places may not become part of your Emory medical record. Also, if you decide to be in this study, it is up to you to let your other health providers know.

In Case of Injury

If you get ill or injured from being in the study, or if new findings result from the study that require additional medical care, Emory will help you get medical treatment. Emory and the study supporter have not, however, set aside any money to pay you or to pay for this medical treatment. The only exception is if it is proven that your injury or illness is directly caused by the negligence of an Emory or study supporter employee. “Negligence” is the failure to follow a standard duty of care.

If you become ill or injured from being in this study, your insurer will be billed for your treatment costs. If you do not have insurance, or if your insurer does not pay, then you will have to pay these costs.

If you believe you have become ill or injured from this research, you should contact Dr. Sorscher or Dr. Hunt. You should also let any health care provider who treats you know that you are in a research study.

Costs
There will be no costs to you for participating in this study, other than basic expenses like transportation, which will be reimbursed after each visit if needed. You will not be charged for any of the research activities. If the study procedures result in any medical complications that would not fall under “injury” as discussed above, the cost of treatment for those complications may be charged to you or your insurance.

Withdrawal from the Study

You have the right to leave a study at any time without penalty.

If you wish to leave the study, you should tell a member of the study staff by phone or in person and they will give you a form to complete (you should return the form to the study staff once it is complete) If you have to mail the form, please send it to:

Department of Pediatrics
Emory University School of Medicine
1768 Haygood Drive, Suite E-280
Atlanta, GA 30322

The researchers also have the right to stop your participation in this study without your consent for any reason, especially if they believe it is in your best interest.

Authorization to Use and Disclose Protected Health Information

The privacy of your health information is important to us. We call your health information that identifies you, your "protected health information" or "PHI." To protect your PHI, we will follow federal and state privacy laws, including the Health Insurance Portability and Accountability Act and regulations (HIPAA). We refer to all of these laws as the "Privacy Rules." Here we let you know how we will use and disclose your PHI for the study.

PHI that Will be Used/Disclosed:

The PHI that we will use or share for the research study includes:

- Medical information about you including your medical history and present/past medications
- Results of exams, procedures and tests you have before and during the study
- Laboratory test results

Purposes for Which Your PHI Will be Used/Disclosed:

We will use and share your PHI for the conduct and oversight of the research study. We will use and share your PHI if needed to provide you with treatment, payment for treatment, and to conduct normal business operations. We may share your PHI with other people and places that help us conduct or carry out the study, such as laboratories, data management centers, Institutional Review Boards (IRBs) and other study sites. If you leave the study, we may use your PHI to determine your health, vital status or contact information.

Use and Disclosure of Your Information That is Required by Law:

We will use and disclose your PHI when we are required to do so by law. This includes laws that require us to report child abuse or abuse of elderly or disabled adults. We will also comply with legal requests or orders that require us to disclose your PHI. These include subpoenas or court orders.

Authorization to Use PHI is Required to Participate:

By signing this form, you give us permission to use and share your PHI as described in this document. You do not have to sign this form to authorize the use and disclosure of your PHI. If you do not sign this form, then you may not participate in the research study. You may still receive non-research related treatment.

People Who will Use/Disclose Your PHI:

The following people and groups will use and disclose your PHI in connection with the research study:

- The Principal Investigator and the research staff will use and disclose your PHI to conduct the study.
- Emory may use and disclose your PHI to get payment for study related treatment and to run normal business operations.
- The Principal Investigator and research staff will share your PHI with other people and groups to help conduct the study or to provide oversight for the study.
- The study supporters may use and disclose your PHI to make sure the research is done correctly and to collect and analyze the results of the research. The study supporters may disclose your PHI to other people and groups like study monitors to help conduct the study or to provide oversight for the study.
- The following people and groups will use your PHI to make sure the research is done correctly and safely:
  - Emory offices that are part of the Human Research Participant Protection Program and those that are involved in study administration and billing. These include the Emory IRB, the Emory Research and Healthcare Compliance Offices, and the Emory Office for Clinical Research
  - Government agencies that regulate the research including: Office for Human Research Protections; Food and Drug Administration
  - Public health agencies
  - Research monitors and reviewers
  - Accreditation agencies

Sometimes a Principal Investigator or other researcher moves to a different institution. If this happens, your PHI may be shared with that new institution and their oversight offices. PHI will be shared securely and under a legal agreement to ensure it continues to be used under the terms of this consent and HIPAA authorization.

Expiration of Your Authorization
Your PHI will be used until this research study ends.

Revoking Your Authorization
If you sign this form, at any time later you may revoke (take back) your permission to use your information. If you want to do this, you must contact the study team at:

Department of Pediatrics
Emory University School of Medicine
1768 Haygood Drive, Suite E-280
Atlanta, GA 30322

At that point, the researchers would not collect any more of your PHI. But they may use or disclose the information you already gave them so they can follow the law, protect your safety, or make sure that the study was done properly and the data is correct. If you revoke your authorization, you will not be able to stay in the study.

Other Items You Should Know about Your Privacy
Not all people and entities are covered by the Privacy Rules. HIPAA only applies to health care providers, health care payers, and health care clearinghouses. If we disclose your information to people who are not covered by the Privacy Rules, including HIPAA, then your information won't be protected by the Privacy Rules. People who do not have to follow the Privacy rules can use or disclose your information with others without your permission if they are allowed to do so by the laws that cover them.

To maintain the integrity of this research study, you generally will not have access to your PHI related to this research until the study is complete. When the study ends, and at your request, you generally will have access to your PHI that we maintain in a designated record set. A designated record set is data that includes medical information or billing

records that your health care providers use to make decisions about you. If it is necessary for your health care, your health information will be provided to your doctor.

We may remove identifying information from your PHI. Once we do this, the remaining information will not be subject to the Privacy Rules. Information without identifiers may be used or disclosed to other people or organizations for purposes besides this study.

Contact Information

If you have questions about the study procedures, appointments, research-related injuries or bad reactions, or other questions or concerns about the research or your part in it, contact Eric Sorscher or Randy Hunt.

This study has been reviewed by an ethics committee to ensure the protection of research participants. If you have questions about your **rights as a research participant**, or if you have **complaints** about the research or an issue you would rather discuss with someone outside the research team, contact the Emory Institutional Review Board at 404-712-0720 or 877-503-9797 or irb@emory.edu.

To tell the IRB about your experience as a research participant, fill out the Research Participant Survey at

https://tinyurl.com/ycewgkke.

Consent and Authorization

TO BE FILLED OUT BY SUBJECT ONLY

Print your name, **sign**, and **date** below if you choose to be in this research study. You will not give up any of your legal rights by signing this form. We will give you a copy of the signed form to keep.

____

Name of Subject

____ ________________

Signature of Subject (18 or older and able to consent) **Date** **Time**

TO BE FILLED OUT BY STUDY TEAM ONLY

____

Name of Person Conducting Informed Consent Discussion

____ ________________

Signature of Person Conducting Informed Consent Discussion **Date** **Time**

You Are Being Asked to Be in a Research Study

Concise presentation of key concepts

You are being asked to be in a research study. A research study is designed to answer a scientific question. If you agree to be in the study you will be one of 30 people who are being studied at Emory.

Why is this study being done?

This study is being done to answer the questions: Can Trikafta™ improve lung function in certain patients with non-cystic fibrosis bronchiectasis (NCFBE)? Can cells taken from a small piece of skin or blood sample be used to predict which individuals with NCFBE will show lung function benefit from Trikafta™? You are being asked to be in this research study because you have a diagnosis of NCFBE and 1) one mutation in a gene called the cystic fibrosis transmembrane conductance regulator (CFTR), and/or 2) slightly elevated levels of chloride in the sweat.

Do you have to be in the study?

It is your choice to join this research study. You do not have to be in it. Your choice will not affect your access to medical care for your condition. Before you choose, take time to learn about the study.

What do you have to do if you choose to join this study?

If you qualify and choose to join the study, you will participate for approximately 12 weeks (5 office visits and 1 phone visit). The researchers will ask you to do the following:

- Have a physical exam
- Have a blood test to confirm you have one mutation in the CFTR (in some cases your medical record may have sufficient information so that we can omit this task)
- Provide urine for safety laboratory tests
- Take a pregnancy test if you are female
- Take a test to measure how well you breathe (Spirometry)
- Take a Sweat Chloride test
- Have an EKG
- Take orally administered Trikafta™
- Provide blood or skin sample (a punch biopsy)
- Answer questionnaires

ALL of these procedures will be paid for by the study.

How is this study going to help you?
If you are in the study, you will be helping the researchers answer the study question. This study is not designed to benefit you directly. Your NCFBE may improve while you are in this study but it may not, and it may even get worse. This study will also learn more about whether cells from your skin or blood can be used to predict which patients with NCFBE might respond best to new drug therapies. The study results may be used to help others in the future.

What are the risks or discomforts you should know about before deciding?
The study will take time. The drug that is being tested may not work any better than regular care and may even cause harm. All studies have some risks. Some risks are relatively small, like being bored or losing time. Some are more serious. Risks for this study include:

- risks of the study drug Trikafta™, some of which include headache, upper respiratory tract infection, abdominal pain, diarrhea and rash
- blood draws have the risk of hematoma (bruising) at the needle site and minimal pain due to the venous puncture procedure
- sweat chloride testing: the Macroduct system is used in hospitals and clinics worldwide to perform sweat chloride testing. This is considered a very safe, painless procedure, but rare minor burns have been reported at the electrode site (although participants may show no discomfort during the test). To further minimize risk, all equipment will be properly cleaned and evaluated before and after the procedure by personnel trained in performance of this assay
- small scar from the skin biopsy
- loss of privacy
- breach of confidentiality

You can find a full list of expected risks, their frequency and severity in the section titled "What are the possible risks and discomforts?"

Alternatives to Joining This Study
If you decide not to enter this study, there is care available to you outside of this research study. This includes all aspects of ongoing and standard care with your usual NCFBE clinical team and care center. If you are taking other drugs such as Kalydeco™, Orkambi™ or Symdeko™ that are approved for the treatment of CF, these may overlap and/or offer similar

benefit to Trikafta™, and you will not be eligible for participation in the trial. You do not have to be in this study to be treated for NCFBE.

Costs

There will be no costs to you for participating in this study, other than basic expenses like transportation. You will not be charged for any of the research activities.

There is more information in the "Costs" section further below.

What Should You Do Next?

Read this form, and/or have it read to you. Make sure the study doctor or study staff explains the study to you. Ask questions such as how much time you will have to spend on the study, any words you do not understand and more details about study procedures. Make sure you understand which parts of the study are research and which are standard care that you would have even if you did not join the study. Take time to think about this and talk about it with your family and friends.

Emory University
Consent to be a Research Subject / HIPAA Authorization

Title: Evaluating Trikafta for the treatment of patients with non-cystic fibrosis bronchiectasis (NCFBE) (Main study)

IRB #: 00004736

Principal Investigators: Eric Sorscher, MD, Department of Pediatrics; Randy Hunt, MD, Department of Medicine; Colin Swenson, MD, Department of Medicine

Study-Supporter: The Marcus Foundation

Introduction

You are being asked to be in a medical research study. This form tells you what you need to think about before you choose if you want to join the study. **It is your choice. If you choose to join, you can change your mind later and leave the study.** Your choice will not cause you to lose any medical benefits. If you choose not to join this study, your doctor will still treat you.

Before you decide:

- Read this form and/or have it read to you
- Listen to the study doctor or study staff explain the study to you
- Ask questions about anything that is not clear

You will get a copy of this form. Take your time to think about joining the study. You may wish to discuss it with family or friends. Do not sign this form if you still have questions or something does not make sense to you. By signing this form, you will not give up any legal rights.

A description of this clinical trial will be available on http://www.ClinicalTrials.gov, as required by U.S. law. This Web site will not include information that can identify you. At most the Web site will include a summary of the results. You can search this Web site at any time.

What is the purpose of this study?

Non-cystic fibrosis bronchiectasis (NCFBE) is a chronic, progressive lung disorder characterized by shortness of breath, wheezing, persistent cough, excessive phlegm production and recurrent lung infections. NCFBE is similar to cystic fibrosis (CF) lung disease – but patients with NCFBE do not exhibit all of the features of CF. Trikafta™ is an oral drug that improves lung function in CF. Trikafta™ is approved for patients with CF but has not been studied in patients with NCFBE. The purpose of this trial is to learn whether Trikafta™ can improve lung function in certain patients with NCFBE. We will also learn whether cells taken from a small piece of skin or blood sample can be used to predict which individuals with NCFBE will show lung function benefit from Trikafta™. We believe patients with NCFBE who have: 1) one mutation in a gene called the cystic fibrosis transmembrane conductance regulator (CFTR), and/or 2) slightly elevated levels of chloride in the sweat will be most likely to exhibit improved lung function on Trikafta™.

This is a pilot study since it is the first time we are testing this drug to learn if and how it helps patients with NCFBE. It is also the first time that cells taken from the skin or blood will be tested as a way to predict whether patients with NCFBE will respond to a drug therapy such as Trikafta™.

What will you be asked to do?

If you qualify for the study, you will receive orally administered Trikafta™ (elexacaftor 100 mg/tezacaftor 50 mg/ivacaftor 75 mg (2 pills once daily in the morning) and ivacaftor (150 mg) once daily in the evening). The drug should be taken with a regular meal of your choice. You will take the medicine for four weeks.

After you take the medicine for four weeks, you will return to our office 4 weeks later for a follow up visit.

You will need to take part in 5 office visits and 1 telephone visit. If you are entered and complete the entire study, you will be in the study for about 3 months. We hope to enroll around 30 participants who are ages 18 years old and older at Emory with a diagnosis of NCFBE. The following tests and procedures will be done at each study visit. All procedures will be explained later in the consent and again in detail by the study Doctor.

Screening (28 Day Screening Period): this visit will take approximately 4-6 hours. We will discuss the study with you and answer any questions. After signing the consent form, the following procedures will be performed. During this visit about 1-2 tablespoons of blood will be collected from a vein in your arm.

- Medical History including your past lung health, previous diagnosis, etc
- Vital Signs including oxygen (O_2) Saturation
- Medication Review
- Physical Exam, the study Doctor will listen to your heart, lungs, etc
- CFTR Genotyping (to confirm that you have a CFTR mutation. This information can sometimes be obtained instead from the medical record)
- Safety laboratory test/Urinalysis
- Serum Pregnancy Test for Females who can become pregnant
- Spirometry
- Sweat Chloride Collection
- EKG
- Questionnaire

Day 1: this visit will take approximately 4-6 hours. During the visit about 1-2 tablespoons of blood will be drawn. The following procedures will be performed.

- Vital Signs including O_2 Saturation
- Adverse Event Review
- Interval Medical history
- Medication Review
- Physical Exam, the study Doctor will listen to your heart, lungs, etc
- Spirometry
- Sweat Chloride Collection
- Safety laboratory test/Urinalysis
- Urine Pregnancy Test for females who can become pregnant
- 3 mm Punch Biopsy of skin or 8 ml blood sample (These samples can be obtained any time between Screening Day 1 or Day 56).
- Questionnaire

- Drug Dispensing
- Drug Administration

Day 7 (+/- 2 days) this will be a telephone contact and will take approximately 30 minutes. The following topics will be discussed.

- Adverse Event Review
- Interval Medical history
- Medication Review

Day 14 (+/- 2 days): this visit will take approximately 4-6 hours. The following procedures will be performed. During this visit about 1-2 tablespoons of blood will be drawn.

- Vital Signs including O_2 Saturation
- Adverse Event Review
- Interval Medical history
- Medication Review
- Physical Exam, the study Doctor will listen to your heart, lungs, etc
- Spirometry
- Safety laboratory test/Urinalysis
- EKG
- Sweat Chloride Collection
- Questionnaire
- Drug Dispensing
- Drug Collection
- Drug Administration

Day 28 (+1 / - 2 days): this visit will take approximately 4-6 hours. The following procedures will be performed. During this visit about 1-2 tablespoons of blood will be drawn.

- Vital Signs including O_2 Saturation
- Adverse Event Review
- Interval Medical history
- Medication Review
- Physical Exam, the study Doctor will listen to your heart, lungs, etc
- Spirometry
- Safety laboratory test/Urinalysis
- Sweat Chloride Collection
- Questionnaire
- Drug Dispensing
- Drug Collection
- Drug Administration

Day 56 (+/- 2 days): this visit will take approximately 4-6 hours. The following procedures will be performed. During this visit about 1-2 tablespoons of blood will be drawn.

- Vital Signs including O_2 Saturation
- Adverse Event Review
- Interval Medical history
- Medication Review
- Physical Exam, the study Doctor will listen to your heart, lungs, etc
- Spirometry
- Urine Pregnancy Test for females who can become pregnant
- Sweat Chloride Collection
- 3 mm Punch Biopsy of skin or 8 ml blood sample if necessary to obtain additional cells (These samples may be obtained within a few days before or several days after day 56).
- Safety laboratory test/Urinalysis
- Questionnaire

Additional information about testing in this study is described below:

Questionnaires:
You will be asked questions about the history of your health and NCFBE.

Physical Exam:
This exam will include the following: blood pressure, height, weight, heart rate, temperature, respiration rate, general appearance, general impression of your chest, heart, head, eyes, ears, nose, throat, neck, abdomen, extremities, skin, neurological and any other notes made by the health care provider.

Blood Draw:
About 1-2 tablespoons of blood will be taken from your arm for safety and other parameters at each visit. If donation of a blood sample used to test *in vitro* cell response to Trikafta is chosen, approximately 2 tablespoons of blood will be collected anytime between Screening Day 1 and/or 2 weeks before, during, or after Day 56 visit.

Pregnancy Test:
Females who are pregnant or nursing a child cannot be in this study. If there is a physical possibility of being or getting pregnant, a serum pregnancy test followed by urine pregnancy tests may be given.

Spirometry:
This is a test that measures how well you breathe. You will wear a nose clip and breathe out forcefully into a machine called a spirometer. This machine measures how much air you blow out and how fast it comes out. If possible, do not take bronchodilators (medication that opens up the airways) the morning of each visit. (Depending on which bronchodilators you use, your Study Doctor may tell you not to take them for 12 – 24 hours before each visit.)

Sweat Chloride Test:
In the sweat test, a place on your arm is stimulated with electrodes to produce sweat. The sweat is caught in a collector disc placed on the skin. We will measure chemicals, such as salt, in your sweat. To collect the sweat, 2 probes will be attached to the skin on your arm for 4-5 minutes. A gel-like medicine called pilocarpine is put on the probes and causes the sweat glands to produce more sweat. The probes stay on for about 4-5 minutes, then are removed and replaced by a disc to collect sweat for about 30 minutes. The entire procedure will then be repeated on the other arm.

Punch Biopsy
If you choose to undergo the punch biopsy test, a sample of your skin will be collected by numbing the area with a local anesthetic. The circular area of skin taken will be similar in size to a small round green pea. The biopsy will be about 3 mm in diameter and about 1-2 mm thick. A punch biopsy may be collected once during the study, and may need to be repeated near the last day of the study if additional biopsy material is needed. The biopsy site will then be sewn up, usually with one or two stitches. If you prefer, a blood test may be used instead of the punch biopsy.

EKG
ECGs will be performed to check the electrical activity of your heart. This test is painless and takes about 10 minutes.

How will your study drug be provided?
The study drug that you will take will be dispensed by the pharmacy and delivered to the principal investigator or study team member. The principal investigator or health care providers on his/her research team will provide the study drug to you. If you have questions about the study drug, you should ask the principal investigator or study nurse. You may also call the pharmacy if you have questions about the study drug. The number for the pharmacy is included on your study drug package, if given one.

> Note: The research team for this study includes non-licensed team members who may obtain your consent, or help guide you through the study. There are some kinds of questions only licensed clinicians can answer. For example, these include detailed questions about drug interactions. If you have questions like these, the non-licensed coordinator will ask a licensed study team member to answer your questions.

Who owns your study data and samples?
If you join this study, you will be donating your samples and data. You will not be paid if your samples or data are used to make a new product. If you leave the study, the data and samples that were already collected may still be used for this study.

What are the possible risks and discomforts?
There may be side effects from the study drug or procedures that are not known at this time. Examples of adverse reactions known in subjects are listed below.

The most common (occurring 5% or more) risks and discomforts expected in this study are:

Headache	17%
Upper respiratory tract infection	16%
Abdominal pain	14%
Diarrhea	13%
Rash	10%
ALT increased (a liver test)	10%
Nasal congestion	9%
Blood CPK increased (a muscle test)	9%
Rhinorrhea (runny nose)	8%
Rhinitis (nose irritation)	7%
AST increased (a liver test)	9%
Influenza	7%
Sinusitis	5%

Blood bilirubin increased (a liver test)	5%

High liver enzymes (called ALT or AST) in the blood have been observed in some subjects. Very high levels of these tests could lead to stopping of Study Drug, and these abnormal blood tests may get better after Study Drug is stopped. In some severe cases, high liver enzymes may be shown as a sign of liver injury, and can become permanent and even be life-threatening.

Possible Risks Based on Animal Studies
In a study in which ivacaftor (a drug found in Trikafta^{TM}) was given to newborn rats, cataracts (cloudiness of the lens of the eye) were seen. No cataracts were seen in studies of older animals (rats and dogs) dosed with ivacaftor for longer periods of time. The importance of this finding in humans is unknown.

Drug Interaction Risks (medicines working with or against each other):
Almost all medicines can cause side effects. Many are mild, but some can sometimes become life-threatening, particularly if they are not treated. The combination of the Study Drug and any other medications, dietary supplements, natural remedies, and vitamins could be harmful to you. It is very important that you tell your study Doctor about every medicine, dietary supplement, natural remedy, and vitamin (or change) while you are in the Study. There are certain herbal medications, like St. John's Wort, and certain fruits and fruit juices, such as grapefruit or grapefruit juice, that you must not take during Study. Your study Doctor will review these with you.

Risks of Discontinuing Study Medication:
Once the four-week treatment period is complete, you will no longer receive Trikafta™. There is a chance that your NCFBE will improve when taking Trikafta™. However, your NCFBE symptoms may appear to worsen after you stop taking Trikafta™ as you return to a respiratory baseline.

Potential risks of blood draws: This is the standard medical method used to obtain blood for tests. There is momentary pain at the time the needle is inserted into the vein, but other discomfort should be minimal. In about 10% of the cases there is a small amount of bleeding under the skin which will produce a bruise. The risk of infection is less than 1 in 1,000.

Sweat chloride testing: the Macroduct system is used in hospitals and clinics worldwide to perform sweat chloride testing. This is considered a very safe, painless procedure, but rare minor burns have been reported at the electrode site (although participants may show no discomfort during the test). The risk is less than 1 in 50,000. To further minimize risk, all equipment will be properly cleaned and evaluated before and after the procedure by personnel trained in performance of this assay.

Risks of the punch biopsy: Significant pain, bleeding, or infection are very rare with this type of punch biopsy. Although we may use a topical anesthetic on your skin so that you won't feel anything, you might feel some discomfort (like a scratch) when we give the medicine, and the next day. After your skin is healed, you might have a very small scar.

If you are a woman: to protect against possible side effects of the study drug, women who are pregnant or nursing a child may not take part in this study. If you become pregnant, there may be risks to you, the embryo, or fetus. These risks are not yet known. If you are a woman of childbearing ability, you and the study Doctor must agree on a method of birth control to use throughout the study. If you think that you have gotten pregnant during the study, you must tell the study Doctor immediately. Pregnant women will be taken out of the study.

If you are a man: the effect of the study drug on sperm is not known. To protect against possible side effects, if you are a man and your female partner becomes pregnant during the Study, you should notify the study Doctor right away. If your female partner becomes pregnant, you will need to stop Study Drug immediately.

When you take the study drug home, keep it out of the reach of children or anyone else who may not be able to read or understand the label. Do not let anyone else take the study drug besides you.

It is possible that the researchers will learn something new during the study about the risks of being in it. If this happens, they will tell you about it. Then you can decide if you want to continue to be in this study or not. You may be asked to sign a new consent form that includes the new information if you decide to stay in the study.

Will you benefit from the study?

This study is not designed to benefit you directly. Patients with NCFBE are more likely to show evidence of a CFTR mutation. Approximately 1 of 30 Caucasian Americans without NCFBE exhibit a CFTR mutation. If we identify patients with NCFBE and a CFTR mutation and/or mildly increased sweat chloride, we will be in a position to ask whether some of these patients would be willing to receive Trikafta™ as a potential new treatment. In this way, the study results may help others in the future.

Will you be paid for your time and effort?

The study will cover expenses related to travel, meals, and accommodations for study visits as necessary. Additionally, there will be an incentive payment of $150 for each in-person visit and $30 for the Day 7 telephone call. If an adult subject requires a caregiver, the study may provide travel expenses, meals, and accommodations for them as well, with all payments being prorated. In the event that an unscheduled visit is necessary for multiple repeat assessments or a cutaneous punch biopsy on a different day, a payment of $150 will be given. If the unscheduled visit is only for a single laboratory sample collection, a payment of $30 will be provided.

We are planning to provide compensation to you by a gift card, check, or a personal payment card. The payment card is a prepaid debit card. We load money onto your card electronically every time you need to be paid. The card scheme is run by Greenphire, an independent company specializing in payments for research studies and clinical trials. To issue your card, we need to give Greenphire some of your personal information and banks and other financial institutions can access this information if they need to verify your identity when you use your card. We ask you to allow us to communicate your name, address, date of birth, research study name and Social Security Number to Greenphire and Emory University Department of Finance. If you want to receive e-mail or text alerts when payments are made to you, we will ask you to provide your e-mail or phone number as well. All this information will be stored on computers owned by Greenphire. Greenphire will not have access to any other information collected during this study. Full instructions about using your card are included when we issue it. Please ask if you have any questions or concerns about the card scheme. You can decline payment if you are concerned about confidentiality.

Research payments in cash or cash equivalents that exceed $600.00 per calendar year must be reported to the Internal Revenue Service (IRS) by the University. The level of reimbursement for this study is at a level that the potential exists for the federal tax reporting to the IRS for your participation in this study. To do this, Emory University Department of Finance needs to keep your Social Security Number on file.

What are your other options?

If you decide not to enter this study, there is care available to you outside of this research study. This includes all aspects of ongoing and standard care with your usual NCFBE clinical team and care center. You do not have to be in this study to be treated for NCFBE.

Taking part in this study may make you unable to participate in some other research studies such as those that exclude people who have mutations in CFTR or elevated sweat chloride. You should discuss this with the researchers if you have concerns. You may wish to research other study options at websites like clinicaltrials.gov or ResearchMatch.org.

How will your private information be protected?

Whenever possible, a study number, rather than your name, will be used on study records. Your name and other identifying information will not appear when we present or publish the study results.

Study records can be opened by court order. They also may be provided in response to a subpoena or a request for the production of documents.

Storing and Sharing your Information

We will store all the data and specimens that you provide using a code. We need this code so that we can keep track of your data over time. This code will not include information that can identify you (identifiers). Specifically, it will not include your name, initials, date of birth, or medical record number. We will keep a file that links this code to your identifiers in a secure location separate from the data.

We will not allow your name and any other fact that might point to you to appear when we present or publish the results of this study.

Your data from this study, including your de-identified genetic information, may be shared with the research community at large to advance science and health. Data from this study may be placed into public databases where, in addition to having no direct identifiers, researchers will need to sign data use agreements before accessing the data. We will remove or code any personal information that could identify you before your information is shared. This will ensure that, by current scientific standards and known methods, it is extremely unlikely that anyone would be able to identify you from the information we share. Despite these measures, we cannot guarantee anonymity of your personal data.

We will use your specimens and data only for research. We will not sell them. However, the results of this research might someday lead to the development of products (such as a commercial cell line, a medical or genetic test, a drug, or other commercial product) that could be sold by a company. You will not receive money from the sale of any such product.

Returning Results to Participants/Incidental Findings

After you complete the study visit, we will share your study data with your NCFBE doctor who can help you interpret the results.

Researchers may also learn something new about your underling health condition. This could include determining that you have a diagnosis of cystic fibrosis, a cystic fibrosis related condition, or a risk of developing cystic fibrosis in your family. If you complete the study visit, we will share your study data with your NCFBE doctor who can help you interpret the results.

Medical Record

If you have been an Emory patient before, then you already have an Emory medical record. If you have never been an Emory patient, you do not have one. An Emory medical record will be made for you if an Emory Atlanta provider or facility gives you any services or procedures for this study.

Copies of the consent form/HIPAA authorization that you sign will be put in any Emory medical record you have now or any time during the study.

The results of some study tests and procedures will be used only for research purposes and will not be placed in your medical record. For this study, those items in some cases may include: results of genotyping and sweat tests.

Tests and procedures done at non-Emory places may not become part of your Emory medical record. Also, if you decide to be in this study, it is up to you to let your other health providers know.

In Case of Injury

If you get ill or injured from being in the study, or if new findings result from the study that require additional medical care, Emory will help you get medical treatment. Emory and the study supporter have not, however, set aside any money to pay you or to pay for this medical treatment. The only exception is if it is proven that your injury or illness is directly caused by the negligence of an Emory or study supporter employee. "Negligence" is the failure to follow a standard duty of care.

If you become ill or injured from being in this study, your insurer will be billed for your treatment costs. If you do not have insurance, or if your insurer does not pay, then you will have to pay these costs.

If you believe you have become ill or injured from this research, you should contact Dr. Sorscher or Dr. Hunt. You should also let any health care provider who treats you know that you are in a research study.

Costs

There will be no costs to you for participating in this study, other than basic expenses like transportation, which will be reimbursed after each visit if needed. You will not be charged for any of the research activities. If the study procedures result in any medical complications that would not fall under "injury" as discussed above, the cost of treatment for those complications may be charged to you or your insurance.

Withdrawal from the Study

You have the right to leave a study at any time without penalty.

For your safety, however, you should consider the study doctor's advice about how to go off the study treatment. If you leave the study before the last planned study visit, the researchers may ask you to complete some of the final steps such as lab work as applicable.

If you wish to leave the study, you should tell a member of the study staff by phone or in person and they will give you a form to complete (you should return the form to the study staff once it is complete) If you have to mail the form, please send it to:

Department of Pediatrics

Emory University School of Medicine
1768 Haygood Drive, Suite E-280
Atlanta, GA 30322

The researchers also have the right to take you out of the study without your consent for any reason. They may do this if they believe it is in your best interest or if you do not agree to changes that may be made in the study.

Authorization to Use and Disclose Protected Health Information

The privacy of your health information is important to us. We call your health information that identifies you, your "protected health information" or "PHI." To protect your PHI, we will follow federal and state privacy laws, including the Health Insurance Portability and Accountability Act and regulations (HIPAA). We refer to all of these laws as the "Privacy Rules." Here we let you know how we will use and disclose your PHI for the study.

PHI that Will be Used/Disclosed:
The PHI that we will use or share for the main research study includes:

- Medical information about you including your medical history and present/past medications
- Results of exams, procedures and tests you have before and during the study
- Laboratory test results

Purposes for Which Your PHI Will be Used/Disclosed:
We will use and share your PHI for the conduct and oversight of the research study. We will use and share your PHI if needed to provide you with treatment, payment for treatment, and to conduct normal business operations. We may share your PHI with other people and places that help us conduct or carry out the study, such as laboratories, data management centers, Institutional Review Boards (IRBs) and other study sites. If you leave the study, we may use your PHI to determine your health, vital status or contact information.

Use and Disclosure of Your Information That is Required by Law:
We will use and disclose your PHI when we are required to do so by law. This includes laws that require us to report child abuse or abuse of elderly or disabled adults. We will also comply with legal requests or orders that require us to disclose your PHI. These include subpoenas or court orders.

Authorization to Use PHI is Required to Participate:
By signing this form, you give us permission to use and share your PHI as described in this document. You do not have to sign this form to authorize the use and disclosure of your PHI. If you do not sign this form, then you may not participate in the research study. You may still receive non-research related treatment.

People Who will Use/Disclose Your PHI:
The following people and groups will use and disclose your PHI in connection with the research study:

- The Principal Investigator and the research staff will use and disclose your PHI to conduct the study.
- Emory may use and disclose your PHI to get payment for study related treatment and to run normal business operations.

- The Principal Investigator and research staff will share your PHI with other people and groups to help conduct the study or to provide oversight for the study.
- The study supporters may use and disclose your PHI to make sure the research is done correctly and to collect and analyze the results of the research. The study supporters may disclose your PHI to other people and groups like study monitors to help conduct the study or to provide oversight for the study.
- The following people and groups will use your PHI to make sure the research is done correctly and safely:
  - Emory offices that are part of the Human Research Participant Protection Program and those that are involved in study administration and billing. These include the Emory IRB, the Emory Research and Healthcare Compliance Offices, and the Emory Office for Clinical Research
  - Government agencies that regulate the research including: Office for Human Research Protections; Food and Drug Administration
  - Public health agencies
  - Research monitors and reviewers
  - Accreditation agencies

Sometimes a Principal Investigator or other researcher moves to a different institution. If this happens, your PHI may be shared with that new institution and their oversight offices. PHI will be shared securely and under a legal agreement to ensure it continues to be used under the terms of this consent and HIPAA authorization.

Expiration of Your Authorization
Your PHI will be used until this research study ends.

Revoking Your Authorization
If you sign this form, at any time later you may revoke (take back) your permission to use your information. If you want to do this, you must contact the study team at:

Department of Pediatrics
Emory University School of Medicine
1768 Haygood Drive, Suite E-280
Atlanta, GA 30322

At that point, the researchers would not collect any more of your PHI. But they may use or disclose the information you already gave them so they can follow the law, protect your safety, or make sure that the study was done properly and the data is correct. If you revoke your authorization, you will not be able to stay in the study.

Other Items You Should Know about Your Privacy
Not all people and entities are covered by the Privacy Rules. HIPAA only applies to health care providers, health care payers, and health care clearinghouses. If we disclose your information to people who are not covered by the Privacy Rules, including HIPAA, then your information won't be protected by the Privacy Rules. People who do not have to follow the Privacy rules can use or disclose your information with others without your permission if they are allowed to do so by the laws that cover them.

To maintain the integrity of this research study, you generally will not have access to your PHI related to this research until the study is complete. When the study ends, and at your request, you generally will have access to your PHI that we maintain in a designated record set. A designated record set is data that includes medical information or billing

records that your health care providers use to make decisions about you. If it is necessary for your health care, your health information will be provided to your doctor.

We may remove identifying information from your PHI. Once we do this, the remaining information will not be subject to the Privacy Rules. Information without identifiers may be used or disclosed to other people or organizations for purposes besides this study.

Contact Information

If you have questions about the study procedures, appointments, research-related injuries or bad reactions, or other questions or concerns about the research or your part in it, contact:

- Eric Sorscher or Randy Hunt

This study has been reviewed by an ethics committee to ensure the protection of research participants. If you have questions about your **rights as a research participant**, or if you have **complaints** about the research or an issue you would rather discuss with someone outside the research team, contact the Emory Institutional Review Board at 404-712-0720 or 877-503-9797 or irb@emory.edu.

To tell the IRB about your experience as a research participant, fill out the Research Participant Survey at **https://tinyurl.com/ycewgkke**.

Consent for skin biopsy:

Would you like to provide a skin biopsy or extra blood sample? You may still participate in the study without providing a skin biopsy if you are willing to provide an extra blood sample.
Please write your initials next to your choice below:
_______YES, I would like to provide a skin biopsy
_______YES, I would like to provide an extra blood sample

Consent and Authorization

TO BE FILLED OUT BY SUBJECT ONLY

Print your name, **sign**, and **date** below if you choose to be in this research study. You will not give up any of your legal rights by signing this form. We will give you a copy of the signed form to keep.

____

Name of Subject

____ ____________________

Signature of Subject (18 or older and able to consent) **Date** **Time**

TO BE FILLED OUT BY STUDY TEAM ONLY

____

Name of Person Conducting Informed Consent Discussion

____ ____________________

Signature of Person Conducting Informed Consent Discussion **Date** **Time**
