# Supplementary figures and images for: Evaluating elexacaftor/tezacaftor/ivacaftor (ETI; Trikafta™) for treatment of patients with non-cystic fibrosis bronchiectasis (NCFBE): A clinical study protocol
Source: PLoS One. 2025 Feb 14;20(2):e0316721. doi: 10.1371/journal.pone.0316721 (PMC11828409; doi:10.1371/journal.pone.0316721)

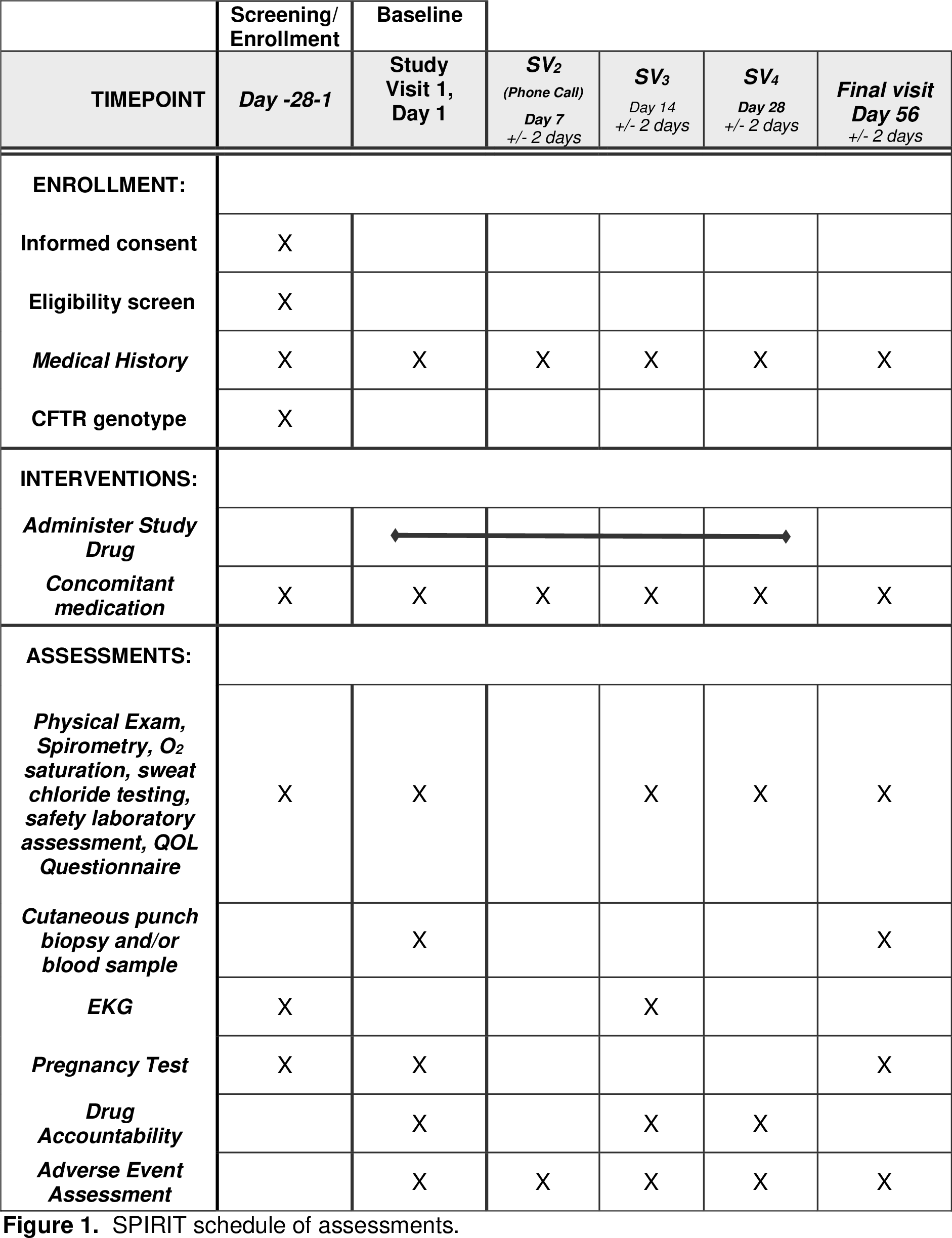

Supplement: S1 Fig — (TIF) [file pone.0316721.s004.tif]
